# A unified framework for potency-oriented AMP discovery via multi-modal learning and guided sequence synthesis

**DOI:** 10.1371/journal.pcbi.1014771

**Published:** 2026-09-03

**Authors:** Wenyu Zhang, Yizheng Wang, Yixiao Zhai, Pinglu Zhang, Yijie Ding, Quan Zou

**Affiliations:** 1 Institute of Fundamental and Frontier Sciences‌, University of Electronic Science and Technology of China, Chengdu, China; 2 Yangtze Delta Region Institute (Quzhou), University of Electronic Science and Technology of China, Quzhou, China; Changsha University of Science and Technology, CHINA

## Abstract

The rapid emergence of drug-resistant pathogens poses a critical threat to global health. With traditional antibiotics losing efficacy, antimicrobial peptides (AMPs) have gained attention for their unique mechanisms and lower resistance potential. We aimed to accelerate AMP discovery by proposing a closed-loop framework that combines AMP-Hunter (a shared-architecture discriminator for AMP classification and MIC prediction that integrates convolutional neural networks with graph neural networks), and AMP-Forge (a generator integrating multiple sequence alignment to select original candidates) and is guided by minimum inhibitory concentration (MIC)for latent space optimization and candidate selection. AMP-Hunter outperformed baseline models in both AMP classification and MIC prediction, achieving 95.82% accuracy and a 95.80% F1 score on the test set for classification, and an R^2^ of 0.9245 with an MAE of 0.2305 for MIC prediction. Guided by its predictions, AMP-Forge generated peptide sequences with lower MIC values and improved physicochemical properties associated with antimicrobial activity. Molecular dynamics simulations further provided in silico evidence supporting the antimicrobial potential of selected sequences by identifying stable membrane disruption and insertion behaviors consistent with membrane-targeting activity. Thus, the generation–screening–validation workflow enables reliable discovery of potent AMPs, and provides a practical strategy for rational peptide design, rapid prediction, and translational applications.

## Author summary

As the efficacy of many traditional antibiotics gradually diminishes, drug-resistant bacteria are posing an increasingly serious threat to global health. Antimicrobial peptides, which are short chains of amino acids capable of combating bacteria, offer a highly promising alternative; however, the process of discovering and designing these peptides is often time-consuming, expensive, and inefficient. To address this challenge, we have developed a novel closed-loop workflow comprising two core components that can be used both to screen and predict peptide activity and to generate more effective novel peptides. Our discriminator accurately identifies promising peptides and predicts their ability to inhibit bacterial growth, outperforming existing methods. Guided by these predictions, the new peptides designed by our generator not only exhibit superior antibacterial activity but also feature improved physical and chemical properties, suggesting improved safety- and stability-related properties. We further confirmed through molecular dynamics simulations that these new peptides effectively disrupt bacterial membranes. Our method accelerates the discovery of effective antimicrobial peptides and provides a practical pathway for peptide design, thereby helping to address the challenge of antimicrobial resistance.

## 1 Introduction

The recent emergence and rapid dissemination of drug-resistant pathogens have resulted in a substantial increase in difficult-to-treat infections, which poses a serious global public health challenge [1,2]. Conventional antimicrobial agents, such as β-lactams and aminoglycosides, have been widely used to treat and prevent bacterial infections. However, their effectiveness has been progressively undermined by two major issues. First, misuse of antibiotics and extensive application of broad-spectrum agents have accelerated the development of drug resistance. Second, common antibiotics used in clinical practice such as penicillin are frequently attributed to causing allergic reactions or intolerance, which restricts treatment options. These factors limit therapeutic efficacy at the individual level and exacerbate the broader public health burden [3,4]. Consequently, the urgent need for alternative or complementary anti-infective strategies that mitigate the drawbacks of conventional antibiotics has become increasingly apparent.

Therapeutic peptides are a promising alternative to conventional antibiotics which are composed of multiple amino acids with specific therapeutic functions. They exhibit lower molecular weight and structural complexity than those of proteins, but they generally show high target selectivity and are less likely to induce immune responses. These characteristics contribute to favorable safety and tolerability profiles [5,6]. Among therapeutic peptides, the antimicrobial peptides (AMPs) form a particularly important subset. Typically AMPs comprise 6–50 amino acid residues, and they exert their antimicrobial effects by disrupting microbial membranes or interfering with metabolic pathways while additionally modulating host immunity; hence they exhibit broad-spectrum activity [7]. Their small sizes and structural flexibility enable AMPs to bind precisely to biological targets and elicit potent antimicrobial responses with notable advantages over conventional small-molecule drugs [8]. Unlike antibiotics with single sites of action, AMPs are considerably less prone to inducing resistance. Nevertheless, few AMPs have reached clinical or commercial application, largely due to slow, costly, and risk-laden experimental discovery pipelines, highlighting the need for high-throughput and accurate computational methods for AMP identification and design.

Deep learning has become a powerful approach for AMP prediction and design by learning discriminative sequence features to improve screening accuracy and alleviate the burden of experimental validation [9]. A wide range of architectures such as CNNs, recurrent models such as RNNs and LSTMs, self-attention mechanisms in Transformer-based models, and protein language models have been applied to AMP research. In particular, AMPlify [10] enhances the performance of computer-based AMP prediction by superimposing two attention mechanisms on bidirectional long-short-term memory layers; however, the small size of the training dataset may make it difficult to distinguish between highly similar positive and negative sequences. iAMPCN [11] has developed a two-stage framework based on multi-sequence encoding and multi-scale CNNs to first identify AMPs and then predict their functional activities. While it outperforms various existing methods on an independent test set, its focus remains primarily on classification-based functional annotation rather than efficacy such as optimizing activity values. Recently, graph neural networks and large-scale protein language models have also been introduced [12,13]. TP-LMMSG [14] achieves unified peptide function prediction by integrating amino acid connectivity graphs, underlying sequence encodings, and protein language model representations into a GAT framework, and outperforms existing state-of-the-art methods on the AMP task in terms of both accuracy and AUC metrics. Beyond AMP-specific prediction, meta-learning has also been explored for peptide discovery under data-limited settings. For example, He et al [15]. proposed a mutual information-based meta-learning framework to accelerate bioactive peptide discovery, highlighting the value of transferable learning strategies for improving peptide identification when labeled data are limited. Besides, variational autoencoders (VAEs), generative adversarial networks (GANs), autoregressive models, and conditional Transformers have been successfully used to generate peptide sequences with desirable properties. Specifically, PepVAE [16] proposes a generative framework that combines a variational autoencoder (VAE) with an antimicrobial activity predictor to generate AMP candidate peptides with high antimicrobial activity and sequence diversity in the latent space. While it outperforms traditional machine learning methods in terms of activity prediction performance, its generative results remain constrained by the scale of the training data and lack sufficient experimental validation and joint optimization of multiple properties, such as peptide stability and toxicity. HydrAMP [17] proposes an AMP design framework that combines deep generative models with conditional optimization strategies, enabling the simultaneous optimization of antimicrobial activity, toxicity, and sequence novelty during the generation process, and has successfully identified multiple peptide candidates with high efficacy against drug-resistant bacteria. Unlike traditional heuristic approaches such as genetic algorithms that generate sequences through random amino acid mutations and are often trapped in local optima, deep learning-based generators use continuous latent spaces to produce more diverse and biologically plausible candidates. Furthermore, the incorporation of conditioning strategies or multi-objective optimization enables these approaches to explicitly guide generation toward sequences with low MIC values and reduced toxicity [18], which accelerates the discovery of potential AMPs [19,20]. In parallel, safety-related peptide properties have also attracted increasing attention. Hasan et al [21]. developed HLPpred-Fuse, a feature-fusion framework for hemolytic peptide prediction, underscoring that toxicity-aware screening is an important complementary step in peptide design pipelines and should be considered alongside activity optimization.

Nevertheless, the use of existing deep learning approaches is constrained by incomplete data metrics, limited feature representation, and narrowly defined prediction tasks during AMP prediction, which result in insufficient generalization and limited applicability. Furthermore, the sequence quality often varies considerably during AMP generation, which leads to poorly balanced antimicrobial efficacy, stability, and safety and absence of rigorous experimental validation. Thus, current studies encounter the following key challenges:

(1) Conventional approaches typically rely on handcrafted features derived from amino acid frequencies or physicochemical properties, which are ineffective in capturing the evolutionary and functional relationships among sequences. Even after combining with deep learning for automatic feature extraction, the limited size of the available datasets restricts the representational power of learned features [22].
(2) Most AMP datasets only provide categorical labels, and activity measures such as MIC are often missing. Consequently, several predictive models are confined to binary classification tasks. Even with the incorporation of MIC values, the predictions often lack compatibility and generalization [23].
(3) Current generative frameworks rarely integrate explicit bioactivity constraints such as MIC. Thus, although the generated sequences exhibit high predicted antimicrobial activity, they additionally exhibit undesirable properties such as excessive hydrophobicity or poor solubility, which undermine their translational potential [24].
(4) Although existing models are capable of producing large numbers of candidate sequences, several studies are confined to theoretical analysis without systematic validation. Thus, the absence of physicochemical property screening and molecular dynamics simulations causes significant uncertainty regarding the true antimicrobial activity and biosafety of the generated peptides [25].

To address these challenges, we have proposed a closed-loop framework that integrates sequence generation, performance screening, and dynamic validation. During the sequence generation process, multiple sequence alignment (MSA) and AMP-Hunter work in close coordination to optimize the sequences. Specifically, MSA is first used to identify and retain functionally conserved fragments typical of antimicrobial peptides, thereby restricting the generation process to biologically meaningful sequence regions. Subsequently, under the guidance of AMP-Hunter, the remaining variable regions are optimized in the latent space. As a discriminator, AMP-Hunter’s predicted MIC values serve as a component of the loss function during generator training, guiding the predictor to more accurately predict sequence MIC values directly from latent variables. Simultaneously, during the generation process, these values guide the evolution of sequences toward lower MIC values. Through this interaction, AMP-Hunter provides activity-driven feedback, guiding sequence optimization toward candidate sequences with stronger antimicrobial efficacy and favorable physicochemical properties, while MSA maintains functional consistency and sequence stability during the generation process.

The contributions of this study are summarized as follows:

(1) To address the limitations of manually designed features and traditional sequence encoders in biological representation capabilities, we used the protein language model ESM2 [26] to extract sequence embeddings as node features and constructed a biologically informed graph structure using the PAM250 evolution matrix. By separately modeling the different AMP classes within the graph neural network and fusing their representations, the discriminator achieves a highly comprehensive and biologically plausible feature representation.
(2) To address the current lack of compatibility between antimicrobial peptide classification and minimum inhibitory concentration prediction tasks, AMP-Hunter is designed as a task-specific discriminator capable of predicting antimicrobial potential and MIC values within a unified framework. Unlike conventional GNN-based approaches that rely on structural inputs [27], this method relies solely on sequence data but consistently outperforms mainstream methods in both classification and regression tasks.
(3) To address the lack of controllability and missing biological constraints in existing antimicrobial peptide generation methods, we developed AMP-Forge which retains functional fragments through multiple sequence alignment while optimizing variable regions in the latent space under MIC constraints. In combination with physicochemical property prediction tools, this ensures that the generated sequences exhibit enhanced antimicrobial potential and maintain favorable safety and stability profiles.
(4) To address the lack of systematic biological validation in many computational antimicrobial peptide studies, we integrated physicochemical screening with molecular dynamics simulations to evaluate the membrane interaction behavior and structural disruption effects of the generated peptides, thereby enhancing the biological reliability of the proposed framework and validating the complementary roles of the generator and discriminator in the “generation–screening–validation” loop.

## 2 Methods

### 2.1 Feature extraction of antimicrobial peptide sequence for graph construction

**Fig 1:**
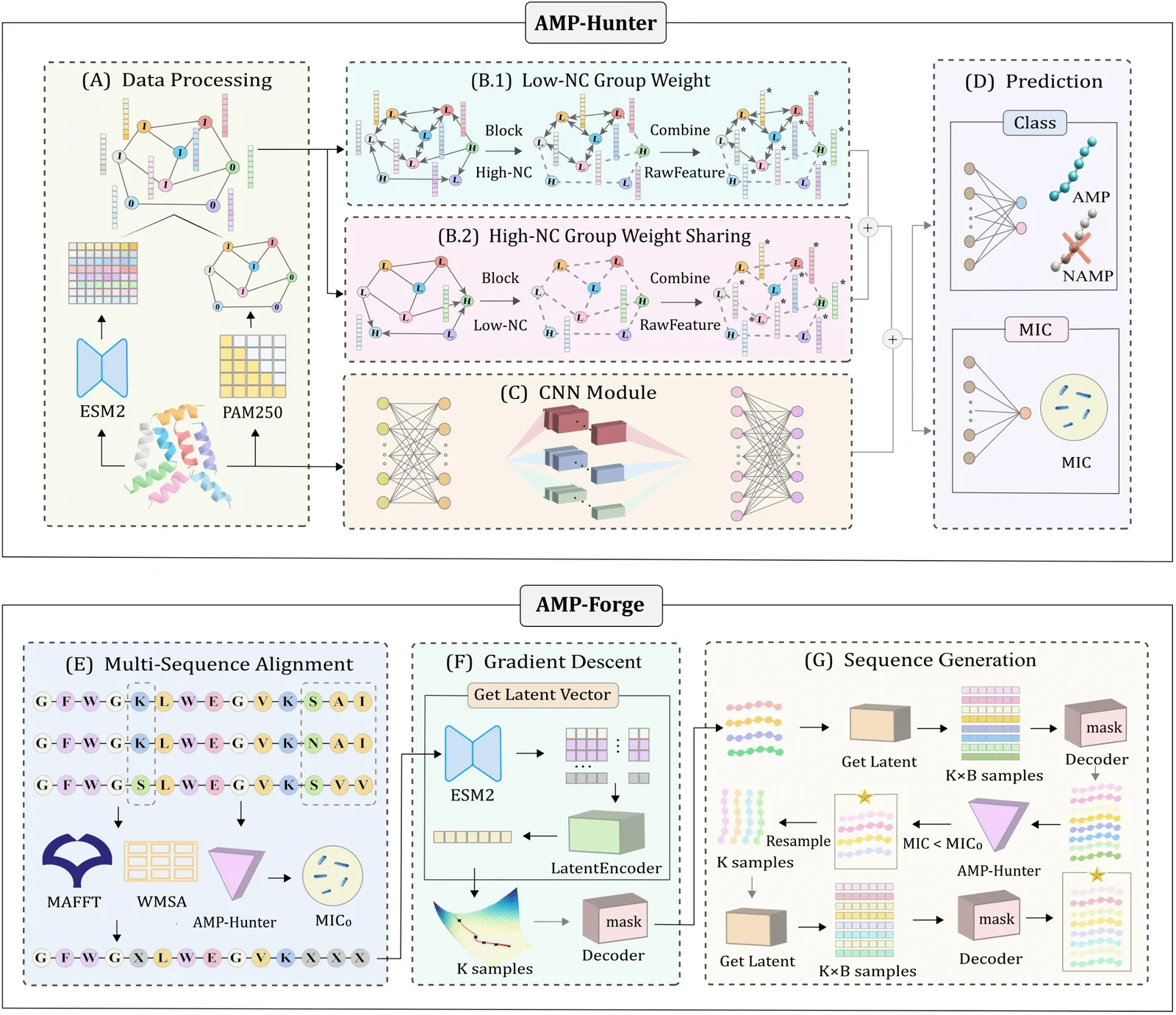
Workflow of the AMP-Hunter and AMP-Forge. **(A)** Data Processing: Graph construction using ESM2 node features and PAM250 edges. (B.1, B.2) GCN Modules: Masking strategies enable information sharing within low‑NC and high‑NC groups. **(C)** CNN Module: Three different-sized 2D convolution kernels and two linear layers were used to extract local features. **(D)** Prediction: Embeddings from GCN and CNN were fused and used in two task-specific prediction settings: category and MIC value prediction. **(E)** MSA Module: Performing multiple sequence alignment, using ‘X’ to mask the unmatched segment, initial MIC predicted by AMP-Hunter. **(F)** Gradient Descent: LatentEncoder encodes vector, performing gradient descent to obtain the optimized sample and decoding via pre-trained decoder **(G)** Sequence Generation: For each latent vector obtained from the gradient descent, B candidate sequences were decoded. Only candidates with predicted MIC values lower than the reference MIC_0_ were retained. The retained samples were resampled for B times and duplicates were removed.

We used ESM2 (esm2_t33_650M_UR50S) [26] to extract sequence features, applied average pooling to obtain fixed-length 1280D vectors as node features to capture structure-function and inherent biological evolutionary information in the sequences for the NCGCN module. Instead of traditional graph construction based on amino acid frequency or physicochemical properties, we built the adjacency matrix using the PAM250 evolutionary matrix which characterizes the likelihood of substitution between amino acids at a certain evolutionary distance to better reflect evolutionary and functional similarities between AMP sequences, with higher scores indicating stronger evolutionary or functional similarity between the sequences. To construct the graph, pairwise similarities between peptide sequences were computed using local sequence alignment with the PAM250 substitution matrix, as implemented by the Smith-Waterman algorithm. This strategy allows sequences of different lengths to be compared without requiring equal-length alignment. For a pair of sequences i and j, we first calculated the raw alignment score S_ij_. To improve comparability across peptide pairs with different lengths, the raw score was further normalized by the geometric mean of the corresponding self-alignment scores. The resulting normalized similarity score was then used for graph construction. The pairwise matching of all sequences (two by two), generated a similarity matrix $S\in R^{N\times N}$, where N indicates the number of AMP sequences. This matrix acts as the basis for subsequent graph structure construction. To generate biologically meaningful and moderately sparse graph structures, we applied a threshold screening strategy to the similarity matrix. In the case where the similarity score between two sequences was higher than the set threshold 𝜏, an edge was established between the corresponding nodes. The threshold was set based on experimental experience to ensure graph connectivity and suppress noise from weakly correlated sequence connections. The graph-construction threshold 𝜏 was selected based on validation performance. We further evaluated 𝜏 values from 0.1 to 0.9 under the same training-validation-test split for both classification and regression tasks, the results for different thresholds are provided in the Appendix S1, S2, S3 and S4 Tables and finally chose 0.2 as the threshold, which provided the best overall and most consistent performance across tasks. Fig 1 shows the complete workflow of the discriminator AMP-Hunter and generator AMP-Forge.

### 2.2 Neighborhood confusion-guided graph convolutional network module

Graph Neural Networks (GNNs) model graph data via node, edge propagation and aggregation. Graph convolutional networks (GCNs), a typical GNN variant, introduce convolutional operations based on neighboring features and locally aggregate and update features in the node feature space. GCNs achieve layer-by-layer diffusion and fusion of node representations by multi-layer stacking to effectively capture higher-order neighbor information. In the construction of graph structures, nodes are not always connected only to homogeneous nodes, and may have edges with heterogeneous nodes as well. However, the non-distinguishing of homogeneous and heterogeneous nodes leads to deep confusion between the nodes, which is not conducive for subsequent classification. To broaden the applicability of the node metric and enhance the fusion effect of local and global features in the graph structure by combining the features of the node with the context information of the neighboring node, this study adopted and optimally extended the Node- and Context-Guided Graph Convolutional Network (NCGCN) module proposed by Zhou [28] et al. for further integrating the relationships between AMPs and extracting discriminative features, which was applied to subsequent classification and regression tasks. NCGCN proposed a new node separation metric known as domain confusion NC, which only considers the neighborhoods that are directly connected to the nodes:


$$\begin{array}{c} {NC}_{i}=-\frac{\text{1}}{\text{log}|C|}\cdot log\frac{|\{v_{j}|v_{j}\in V_{i}^{\text{1}}\cup \{v_{i}\},\;y_{i}=c_{max}\}|}{|V_{i}^{\text{1}}\cup \{v_{i}\}|} \end{array}$$ (1)

where C represents the number of node categories and $c_{max}$ denotes the category to which the majority of nodes in the neighborhood belong. It focused on the most common label in the neighborhood of $v_{i}$. When the neighborhood shows a category with a significantly larger number of nodes, i.e., when the NC value is smaller than the selected threshold, the classification problem of the current node is considered relatively easy to handle. The graph was constructed using only sequence-derived features and PAM250-based similarity scores, without using any class labels or MIC values. During optimization, supervised loss was computed exclusively on training nodes. The NC mask was updated using forced labels for training nodes and predicted labels for non-training nodes. Specifically, after each update step, ground-truth labels were retained for training samples, whereas validation and test samples were assigned their current predicted labels before recomputing the NC-based partition. Therefore, validation and test labels were never used in graph construction or parameter optimization.

However, the original definition is only applicable to the classification task and cannot be directly used in the regression scenario where the node labels are continuous real values. For this reason, this study proposes a new NC calculation method for the regression task of predicting the MIC value of AMPs to adapt the consistency discrimination under continuous labels. By introducing a local consistency metric between consecutive labels, the NC metric was flexibly applied to the regression task, which extended the scope of application of the original NCGCN model and improved the relevance and effectiveness of node feature learning. Specifically for the regression task, the NC metric of a node was defined as the absolute difference between its label value and the mean value of its neighboring labels:


$$\begin{array}{c} {NC}_{i}=|y_{i}-\frac{\sum_{v_{j}\in V_{i}^{\text{1}}}y_{j}}{|V_{i}^{\text{1}}\cup \{v_{i}\}|}| \end{array}$$ (2)

where, $v_{j}$ indicated the neighboring node of $v_{i}$; when the MIC value of the node is close to the mean value of its neighbors, it indicates high consistency for the node. In contrast, if the NC value is large, it indicates that the node features deviate from the neighboring features and belongs to low consistency node.

The network framework of NCGCN primarily comprises two sets of two-layer GCNs that are independent of each other. All the nodes in the graph are distinguished as nodes in the low NC group and nodes in the high NC group according to the NC metric. The low NC node group was trained in a two-layer GCN network, whereas the high NC node group was trained in a different independent two-layer GCN network with no weight sharing between each other. The first layer GCN performed a target masking operation on the adjacency matrix A of the graph and only retained the rows corresponding to the nodes in the same group to prevent the nodes in the other group from receiving information from the nodes in this group. For example, the low NC group can be expressed using the following equation as:


$$\begin{array}{c} H_{\text{1}}^{low}=\sigma (Norm(M^{low}A)\cdot XW_{\text{1}}^{low}) \end{array}$$ (3)

where, $H_{\text{1}}^{low}$ indicates the output feature matrix of the low NC group in the first GCN layer; $W_{\text{1}}^{low}\in R^{d\times d_{\text{h}}}$ indicates the weight of the low NC group in the first layer. The diagonal matrix $M^{low}$ is the mask matrix of the low NC group. The first layer network construction for the high NC group is similar.

The second GCN layer filters out the connection with nodes belonging to other groups for message separation delivery by performing source mask operation on the adjacency matrix, which ensures that the nodes would only aggregate the information from the same group to get further feature outputs. The formula for the second layer implementation of GCN for the low NC group is expressed as:


$$\begin{array}{c} H_{\text{2}}^{low}=\sigma (Norm({AM}^{low})\cdot H_{\text{1}}^{low}W_{\text{2}}^{low}) \end{array}$$ (4)

Improvement and conversion of GCN by separating the different NC groups through source mask operation and target mask operation enabled the optimization of different node groups to be independent of each other and prevented unreasonable mixing of information. To retain the important information in the original features and portray the node characteristics in a comprehensive manner, the original features were introduced by linear variation, and the original features and GCN output features were fused using learnable scalar weights.


$$\begin{array}{c} H_{\text{3}}^{low}=\omega^{low}\cdot H_{\text{2}}^{low}+(\text{1}-\omega^{low})\cdot (XW_{x}) \end{array}$$ (5)

Where *X* is the original feature matrix, $W_{x}\in R^{d\times d_{\text{h}}}$ is the original feature weight matrix.

By combining the output feature matrix with the original features, the details in the original features were preserved to avoid the loss of information. The original features reflect the attributes of the nodes themselves, whereas the GCN output feature matrix incorporates the topological structure information and feature information of the nodes and their neighbors. Combining the two enhanced the ability of the model to express the node features. It simultaneously improved the generalizability of the model, which would help avoid high sensitivity to the structure of the graph or high disturbance from noise. Finally, the feature embedding of the NCGCN module that integrates the local neighborhood features of the nodes and captures higher-order global structural information through multilayer propagation was obtained by fusing the low and high NC groups of the features that incorporate the original features and the GCN output features.

### 2.3 Feature fusion of CNN and NCGCN for antimicrobial peptides prediction

In this study, a lightweight yet feature-rich CNN architecture was employed to process AMP sequence representations. Peptide sequences were first encoded using one-hot encoding and projected for initial feature mapping. Three 2D convolutional kernels of different sizes k∈{2,3,4}, each with size k×h and 100 channels were used for local feature extraction, the output of different convolutions are spliced. To complement the local feature extraction capability of CNNs, we integrated NCGCN to model global sequence relationships from ESM2-derived graphs. The CNN and graph features were fused to form a shared feature representation. Based on this shared backbone, AMP-Hunter was implemented in two task-specific settings: one for AMP classification and the other for MIC regression. This fusion strategy effectively uses the respective advantages of the CNN and NCGCN to enhance the overall performance of the model in the AMP prediction task.

Additionally, the CNN and NCGCN fusion framework proposed in this study exhibits a high degree of task compatibility in terms of structural design. Algorithm 1 shows the workflow of AMP-Hunter. By reasonably and separately designing the task-specific output layer and the loss function, we could flexibly switch between the classification task and regression task with almost no need for altering the internal structures of the CNN and NCGCN modules. This treatment improved the generality and scalability of the framework, which enabled the same core feature-extraction architecture to be transferred across related AMP prediction tasks while maintaining stable feature representations.

Algorithm 1: Workflow of Discriminator AMP-Hunter

Input: antimicrobial peptides dataset${\{X,Y,MIC\}=\{x_{i},y_{i},{mic}_{i}\}}_{i=\text{1}}^{N}$, pre-trained ESM2 model $\varphi_{ESM\text{2}}$, NCGCN training model $\vartheta_{NCGCN}$, edges matrix S, threshold for classifying high and low NC values thresh, partition times T, dataset division ratio ratio, iteration number R, patience to termination P, CNN model $\vartheta_{CNN}$

1: $X^{embed}\leftarrow \varphi_{ESM\text{2}}(X)$

2: $S=PAM\text{2}\text{5}\text{0}(X^{embed})$

3: ${NC}_{i}\leftarrow \text{0},i=\text{1},\text{2},...,N$

4: for t=1 to T do

5: $M^{low},M^{high}\leftarrow J_{N\times N}$

6: Best←0

7: $X^{train},X^{val},X^{test}\leftarrow split(X^{embed},S,ratio)$

8: for epoch=1 to R do

9: $\text{train}(\vartheta_{NCGCN},M^{low},M^{high},X^{train})$

10: $E_{val}\leftarrow \vartheta (X^{val},M^{low},M^{high})$

11: $\text{if }{\text{E}}_{\text{val}}\text{ is better than Best then}$

12: $Best\leftarrow E_{val}$

13: $E_{test}\leftarrow \vartheta (X^{test},M^{low},M^{high})$

14: if task="classification" then

15: $Y^{pred}\leftarrow \vartheta_{NCGCN}(X^{embed},NC,M^{low},M^{high},S)$

16: ${NC}_{i}\leftarrow f_{NC}(Y^{pred},S)$

17: end if

18: if task="regression" then

19: ${MIC}^{pred}\leftarrow \vartheta_{NCGCN}(X^{embed},NC,M^{low},M^{high},S)$

20: ${NC}_{i}\leftarrow f_{NC}({MIC}^{pred},S)$

21: end if

22: $M^{low},M^{high}\leftarrow f_{mask}(NC,thresh)$

23: end if

24: $\text{if Best is better than}{\text{E}}_{\text{val}}\text{for P epoch then}$

25: Break

26: end if

27: $H^{NCGCN}\leftarrow \vartheta_{NCGCN}(X^{embed},NC,M^{low},M^{high},S)$

28: end for

29: $H^{CNN}\leftarrow \vartheta_{CNN}(X)$

30: $H\leftarrow \text{concat}(H^{CNN},H^{NCGCN})$

31: $E_{train},E_{val},E_{test}\leftarrow FC(H)$

32: end for

### 2.4 Framework of generator AMP-Forge in sequence generation

Unlike fully de novo peptide generators designed to explore a vast sequence space, AMP-Forge employs a template-based optimization strategy. By preserving conserved regions identified through multiple sequence alignment and applying local sequence evolution based on minimum inhibitory concentration to variable regions, this method is better suited for optimizing known AMP-like motifs into more potent candidates while maintaining biological plausibility and reducing the risk of mutations that lead to loss of activity. To generate new sequences with potential antimicrobial activities, the potential space distribution and elements determining their antimicrobial properties needed to be learned from the known AMP sequences; hence, this study combined the proposed discriminator AMP-Hunter to design a generator AMP-Forge, which retains the important sequence fragments of the original sequences related to the antimicrobial properties, and optimizes and improves the rest of the original sequences to form a complete process from AMP design to activity prediction, while improving the efficiency and automation of AMP development. In AMP-Forge, MIC-guided optimization primarily occurs during training, where the generator simultaneously learns sequence reconstruction and the prediction of MIC values from latent variables, with the MIC serving as the supervised regression target for the training loss function. During the generation, the latent representations of parent peptides are iteratively adjusted based on the predicted MIC values, thereby prioritizing candidate sequences with lower predicted MIC values. Subsequently, the optimized latent vectors are decoded into candidate sequences, which are further evaluated by MIC values predicted by AMP-Hunter. Generated sequences with predicted MIC values lower than those of their corresponding parent sequences are retained. Thus, in our framework, MIC serves both as a guidance in the latent space optimization process and as a selection criterion in the directed evolution process. Algorithm 2 shows the workflow for the generator AMP-Forge.

Algorithm 2: Workflow of Generator AMP-Forge

Input: antimicrobial peptide sequence for optimization x, ESM2 encoder with frozen parameters $\varphi_{ESM\text{2}}$, LatentEncoder $\varphi_{latent}$, Decoder θ, Predictor *P*, AMP-Hunter D, iteration number iter, sequences number obtained from gradient descent *k*, candidate number m, random perturbation threshold T

1: $x^{embed}\leftarrow \varphi_{ESM\text{2}}(x)$

2: ${MIC}_{\text{0}}\leftarrow D(x)$

3: for j=1 to k

4: $z_{\text{0}}\leftarrow N(\mu_{\varphi_{latent}}(x^{embed}),\sigma_{\varphi_{latent}}^{\text{2}}(x^{embed}))$

5: obtain latent vectors through gradient descent optimization $\text{z}\leftarrow \text{GD}(z_{\text{0}},P,D(x^{embed}))$

6: $x_{j}^{\prime }\leftarrow \theta (z)$

7: ${x_{j}^{\prime }}^{embed}\leftarrow \varphi_{ESM\text{2}}(x_{j}^{\prime })$

8: end for

9: for i=1 to iter do

10: $X_{i}\leftarrow \emptyset$

11: for j=1 to k do

12: for r=1 to m do

13: $z_{r}\leftarrow N(\mu_{\varphi_{latent}}({x_{j}^{\prime }}^{embed}),\sigma_{\varphi_{latent}}^{\text{2}}({x_{j}^{\prime }}^{embed}))$

14: if random(0,1)>T then

15: $z_{r}\leftarrow z_{r}+(s-\delta *i)\varepsilon \;,\;\varepsilon \sim N(\text{0},I)$

16: end if

17: $x_{r}^{\prime }\leftarrow \theta (z_{r}),\;x_{r,t}^{\prime }=\left\{ \begin{array}{c} @l{\theta (z_{r})}_{t}\;,\;t-positionresidueismasked.\\ x_{t}\;,\;t-positionresidueisnotmasked \end{array}\right.$

18: ${MIC}_{r}\leftarrow AMP-Hunter(x_{r}^{\prime })$

19: $\text{if }{MIC}_{r}<{MIC}_{\text{0}}\text{ then}$

20: $X_{i}\leftarrow X_{i}\cup \{x_{r}^{\prime }\}$

21: end if

22: end for

23: calculate weight $\omega_{r},r=\text{1},\text{2},..,m$ for resample by AMP−Hunter(X)

24: $s\leftarrow {Multinomial(\omega }_{r},X_{i})$

25: for r=1 to m do

26: $z_{s_{r}}\leftarrow N(\mu_{\varphi_{latent}}(s^{embed}),\sigma_{\varphi_{latent}}^{\text{2}}(s^{embed}))$

27: $s_{r}^{\prime }\leftarrow \theta (z_{s_{r}})$

28: $X_{i}\leftarrow X_{i}\cup \{s_{r}^{\prime }\}$

29: end for

30: end for

31: $X_{i}\leftarrow X_{i}\cup X_{i-\text{1}}$

32: $X_{i}\leftarrow \text{BestK}(X_{i})$

33: end for

### 2.5 Training module of the generator AMP-Forge

To learn the potential spatial representations of AMPs, we constructed a generative modeling framework based on variational autoencoder (VAE). The original space of peptide sequences is high-dimensional, discrete, and shows abundant redundant information, which hinders effective modeling. In contrast, learning the potential space distribution using VAE aided in mapping the peptide sequences to a continuous low-dimensional representation space. This compressed and extracted the effective features of the peptide sequences. Additionally, it searched for low-MIC regions by combining with the MIC prediction module for the gradient optimization of the potential space to intelligently generate candidate peptides with smaller inhibitory concentrations without relying on exhaustive enumeration or random sampling. The framework reconstructed the input peptide sequences for training and correlated the structure of the latent space with biofunctionality by mapping the latent variables to the minimum inhibitory concentration. This provides the basis for the subsequent optimization of the generation task.

AMP-Forge integrates an ESM2 encoder (ESM2_t33_650M_UR50S), a variational autoencoder (VAE), and a latent-space MIC predictor. The ESM2 model initially obtained the feature representations $H\in R^{L\times D}$ of the AMP sequences by coding the extracted contextual features of each amino acid residue, where, L indicates the sequence length, and D indicates the feature dimension. Thus the set of alphabetical sequences with discrete amino acid representations were mapped to the continuous feature space. To further integrate the features at each position of the sequence, the model introduced a global attention mechanism, which weighted and summarized the ESM2-encoded sequence representations to obtain a global representation $Z\in R^{D}$ of the sequence. The latent variable encoder $\varphi :\text{ Z}\to {\text{Z}}^{\text{h}}\in {\text{R}}^{\text{h}}$, where h indicates the dimension of the encoded features, used two feed-forward neural networks to compute the mean and variance under logarithm of the sequence features from the global representation vector and sampled the latent variables from a Gaussian distribution using a reparameterization technique. In this manner, the model improved the learning of the sequence distributions by projecting the sequences from the feature space encoded in the ESM2 to the latent space.


$$\begin{array}{c} z_{i}=\mu_{i}+\in \cdot exp(\frac{\text{1}}{\text{2}}\text{log }{\sigma_{i}}^{\text{2}}),\;N(\text{0},I) \end{array}$$ (6)

where $\text{I}\in {\text{R}}^{\text{h}}$, ∈ is a random vector not greater than 1.

To enhance the generation quality of the decoder, the temporal dimension was upsampled via transposed convolution prior to decoding; thus, the latent variables were converted into structured features that were suitable for sequence-level decoding. To decode the latent representation features into antimicrobial peptide sequences, a gated recurrent unit (GRU)-based recurrent neural network decoder $\theta :{\text{Z}}^{\text{h}}\to {\text{X}}^{\prime }\in {\text{R}}^{\text{V}}$ was adopted, where V indicates the size of the amino acid alphabet. The decoder combined the attention mechanism to weight and sum of the upsampled latent variables to generate context vectors. Then, these vectors were spliced with the current GRU outputs and fed into the linear layer to generate the final outputs.

Structured potential representation features that satisfied the Gaussian distribution were constructed by calculating the mean and variance under logarithm of the global representation of AMP sequences. The constraints on the reconstruction error of the potential space and the KL dispersion were combined to reduce the introduction of the zero information, increase the retention of semantic information pertaining to the sequences, and avoid arbitrary encoding in the potential space. However, the peptide sequences generated by the decoder in this manner only restored the original sequences to a large extent, and did not effectively distinguish between regions with high and low fitness. To achieve latent variable representations that are as similar as possible for sequences with similar fitness, the latent variables should retain the information related to the fitness to increase the search efficiency in the potential space. Additionally, a predictor that directly predicts the MIC values from the latent variables was trained. It comprised two linear layers and a dropout layer. The loss value between the predicted MIC and real MIC of the dataset during the training process was reduced to increase the accuracy of predicting the MIC of the sequence of unknown labeled peptides in the optimization session, and at the same time, retain the separability of the range of different fitness. The aim of this training was to minimize reconstruction loss such as the cross-entropy loss, KL dispersion, and mean-square error between the predictor-predicted MIC and actual MICs. This ensures that the latent space is compact and continuous and that the constructed latent variables are close to the standard Gaussian distribution while retaining sufficient useful information regarding the sequences to restore the original sequences. It additionally increases the semantic separability of sequence adaptation values in the latent space, which makes finding the target adaptation value interval during optimization easier.


$$\begin{array}{c} L_{train}=\frac{\rho }{N}\sum_{i=\text{1}}^{N}CE(x_{i},x_{i}^{\prime })+\frac{\beta }{N}D_{KL}(N(\mu_{i},{\sigma_{i}}^{\text{2}})||N(\text{0},I))+\frac{\epsilon }{N}\sum_{i=\text{1}}^{N}(p_{i}-y_{i}) \end{array}$$ (7)

where ρ, β, and ϵ denote the weights of reconstruction, KL divergence, and MIC regression losses, respectively. This joint constraint aligns latent representations with both sequence structure and antimicrobial activity, improving the separability of peptides with different MIC levels and enhancing the efficiency of latent-space search.

### 2.6 Sequence optimization via latent evolution and targeted mutation

Instead of direct random residue mutation, which may disrupt functional motifs, key conserved regions were first identified via multiple sequence alignment MAFFT [29] and WMSA [30]. Sequences were segmented and aligned within groups, and fragments with ≥3 matches were retained as high-confidence regions. Fragments with predicted low MIC values were preferentially spliced to form optimization templates. Unmatched regions were masked (denoted by“X”) and designated for local regeneration, enabling targeted refinement while preserving core functional segments. To avoid trapping generated sequences in regions associated with high MIC values, we used the trained AMP-Hunter to estimate the MIC of each candidate and defined lower predicted MIC values as the training objective for the predictor and the guidance signal for latent space optimization. Gradient descent was then applied in latent space to iteratively update latent vectors toward regions with lower predicted MIC values. MSA and AMP-Hunter perform distinct yet closely coordinated functions. MSA narrows the search space by retaining biologically conserved antimicrobial motifs, while AMP-Hunter guides the optimization of the resulting space by evaluating the antimicrobial potential and MIC of the generated candidate molecules. Specifically, the original sequence was first extracted using the frozen ESM2 to extract features. Then its mean and logarithmic variance were learned from the latent space encoder, followed by the construction of the initial latent variables. Subsequently, the trained predictor was used to predict the MIC values from the latent variable vectors for backpropagation in each iteration, which was combined with a gradient descent optimizer to continuously update the latent variables.


$$\begin{array}{c} z_{i}^{t}=z_{i}^{t-\text{1}}-\eta \cdot \nabla_{z}f(z_{i}^{t-\text{1}}),i=\text{1},\text{2},...,k \end{array}$$ (8)

where ${\text{z}}_{\text{i}}^{\text{t}}$ denotes the latent variable at iteration t, f(·) is the predicted MIC, $\nabla_{\text{z}}\text{f}({\text{z}}_{\text{i}}^{\text{t}-\text{1}})$ is the gradient with respect to the latent variable. Eventually, initial population contained k samples over multiple iterations

To further improve the antimicrobial performance of the generated sequences, after obtaining the initial population sequences based on gradient optimization, a directed evolutionary strategy was introduced to iteratively explore the latent space. Specifically, each sequence in the population was scored using the trained MIC predictor and ranked according to the antimicrobial performance. Subsequently, the population was replicated *m* times, which expanded the population to k×m size, and each sequence was encoded in the latent space. To avoid extremely early population convergence into localized regions, which would lead to the missing out of high-quality potential sequences, we set thresholds for random perturbations.


$$\begin{array}{c} z_{i}\leftarrow z_{i}+(s-\delta *iter)\epsilon ,\;\epsilon \sim N(\text{0},I) \end{array}$$ (9)

where s is the base scaling factor, δ controls the decay of perturbation across iterations. After decoding, candidate sequences were evaluated by the MIC predictor and ranked in ascending order of predicted MIC. The top k candidates with the lowest predicted MIC values were retained for the next iteration.

### 2.7 Mask-preserved decoding and Monte Carlo resampling

To further guide the peptide sequences to evolve in a more optimal direction on the basis of potential space optimization, we proposed a decoding process based on MIC value-guided Monte Carlo sampling [31] with mask retention strategy to maximally preserve the well-behaved segments of the original sequences while locally exploring them. Specifically, multiple sequence alignment was performed in advance for each input sequence to mark the high matching regions that should be retained; additionally, the mask character was used to mark the regions that needed to be optimized. During the generation process, the decoder reconstructed only the segments that were masked, whereas the original content was kept unchanged at amino acid sites that were not masked. Thus, unification of local retention and global performance optimization at the structural level were achieved. For a given sequence in which some of the positions were masked (denoted by the character ‘X’), and the real residues were preserved at the rest of the positions, a binary mask vector ‘m’ was used to implement this positional masking mechanism:


$$\begin{array}{c} m_{t}=\left\{ \begin{array}{c} @l\text{1},\;ifx_{i,t}{=}^{\prime }X\prime \\ \text{0},\;ot\text{h}erwise \end{array}\right. \end{array}$$ (10)

where $x_{i,t}^{out}$ is the t-th residue of the sequence. For the output sequence $x_{i}^{out}$ generated by the decoder from the latent vector $z_{i}$, the formula is expressed as:


$$\begin{array}{c} x_{i,t}^{out}=m_{t}\cdot x_{i,t}+(\text{1}-m_{t})\cdot \hat{x_{i,t}} \end{array}$$ (11)

where $x_{i,t}^{out}$ is the t-th residue finalized by the generating sequence, $x_{i,t}$ is the t-th residue of the input sequence, and $\hat{x_{i,t}}$ is the residue generated by the trained generator.

After obtaining the final generated sequences, a Monte Carlo resampling strategy based on softmin weights was proposed for selecting the highly promising candidate sequences in each round of evolution. The softmin-based resampling scheme assigns larger sampling weights to candidates with lower predicted MIC values, thereby biasing the evolutionary search toward more potent sequences. In each round of iteration, the currently retained set of optimal sequences were subjected to latent variable sampling and sequence reconstruction using the trained AMP-Forge. For each reconstructed sequence, a trained MIC prediction model was used to predict the MIC values of the current candidate sequences. Then, the resampling mechanism was introduced to filter and resample the candidate sequences. For a set of candidate sequences $X_{i=\text{1}}^{N}$, their corresponding MIC prediction values are ${F(X)}_{i=\text{1}}^{N}$, and the formula for weighted sampling is:


$$\begin{array}{c} \omega_{i}=\frac{exp(-f(x_{i}))}{\sum_{j=\text{1}}^{N}exp(-f(x_{j}))},i=\text{1},\text{2},...,N \end{array}$$ (12)

The set of candidate sequences was polynomially sampled according to the calculated weights, which resulted in a new resampled subset:


$$\begin{array}{c} s_{i}=\omega_{i}\cdot x_{i}^{out},i=\text{1},\text{2},...,N \end{array}$$ (13)

where $s_{i}$ indicates the i-th sequence after resampling. Top k ranked sequences were merged with previous remained sequences and iteratively evolved. This fusion-ordering strategy enabled the model to maintain the diversity of the search in continuous iterations while gradually converging to the region of sequences with the best performance.

## 3 Results

### 3.1 Datasets

The datasets used in this study were derived from the curated AMP resources reported by Dean [32] et al. and Wang [12] et al., Sequences containing non-standard amino acids, duplicate sequences, and empty sequences were removed and were ultimately organized separately for AMP classification, MIC regression, and sequence generation.

For AMP-Hunter, we used a dataset containing 3,280 positive AMP entries and 3,280 negative entries. The positive samples were sourced from Dean [32] et al.; each positive AMP entry consists of an AMP sequence and the logarithmically transformed value of its corresponding minimum inhibitory concentration (MIC) in E. coli (all MIC values mentioned below refer to the log-transformed MIC values). The negative samples were sourced from Wang [12] et al.; from which we randomly sampled the same number of negative samples as positive samples. Following the dataset configuration used in the reference study, negative samples were assigned the MIC label 3.9999 during model training in the regression branch. To minimize bias potentially introduced by highly heterogeneous peptide chemical structures and to facilitate subsequent synthesis and validation, we retained only peptides with fewer than 40 residues and containing no cysteine residues or sequence modifications.

For AMP-Forge, we used the AMP generation dataset reported by Dean et al. [30], which contains 6,142 AMP sequences and their corresponding MIC values. All peptide sequences in this dataset range in length from 4 to 40 residues. For subsequent generative analysis, the peptides were further divided into four length intervals (4–9, 10–19, 20–29, and 30–39 residues), enabling the comparison of generated peptides with subsets of original AMPs of matching lengths. This grouping strategy minimized the confounding effects of peptide length on comparisons of motifs, physicochemical properties, and sequence patterns, thereby enhancing the interpretability of the generative results.

**Fig 2:**
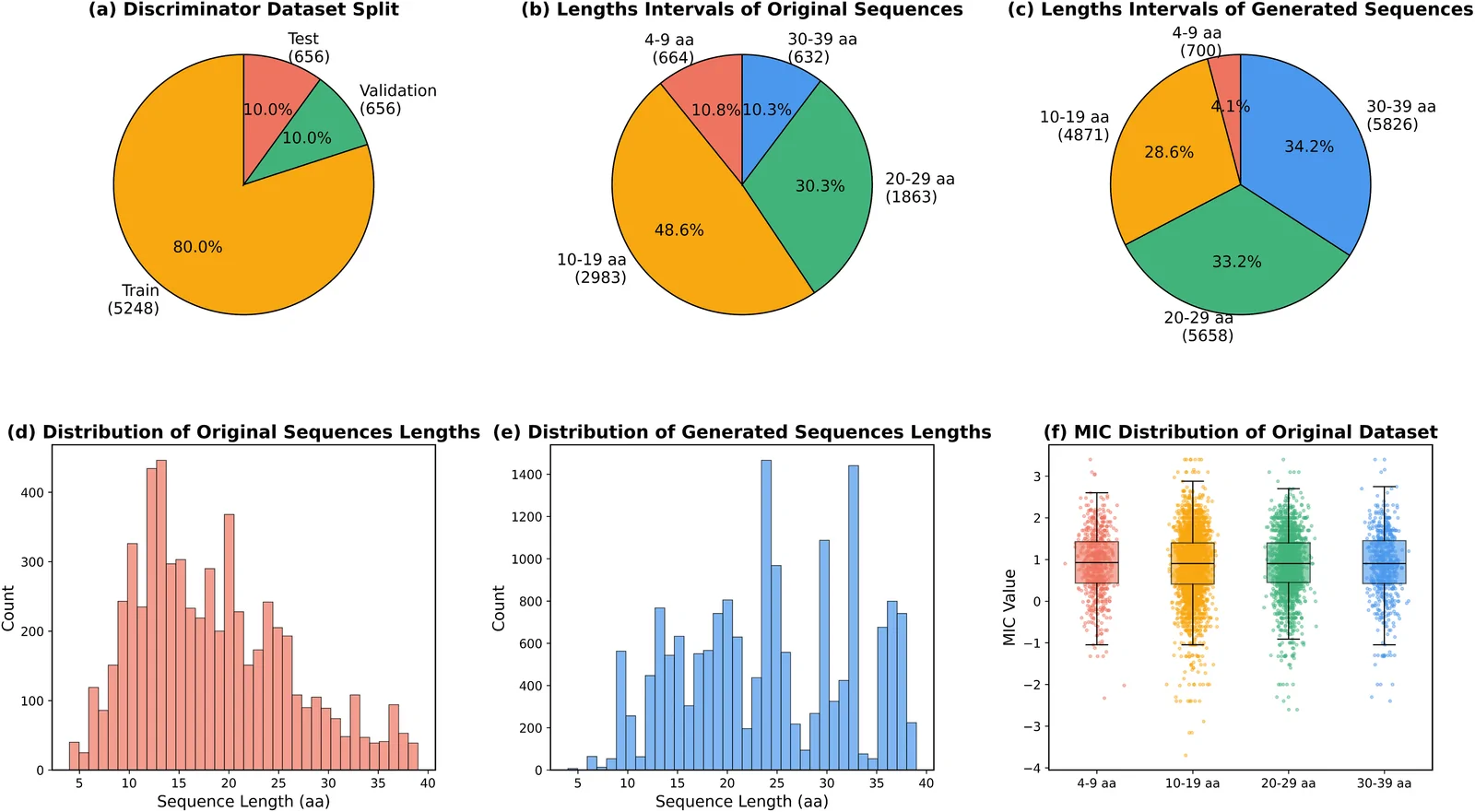
Dataset split and length information: (a) Discriminator Dataset Split: Data Split for the AMP-Hunter Classification and Regression Tasks. **(b)** Lengths Intervals of Original Sequences: The number of sequences in the length range of the datasets used by AMP-Forge. **(c)** Lengths Intervals of Generated Sequences: The number of sequences in the length range of the sequences generated by AMP-Forge. **(d)** Distribution of Original Sequences Lengths: Length distribution of the dataset used by AMP-Forge. **(e)** Distribution of Generated Sequences Lengths: Length distribution of the sequences generated by AMP-Forge. **(f)** MIC Distribution of Original Dataset: MIC Distribution of the dataset used to generate the task.

Information relevant to the dataset such as size, dataset split, length distribution of the original and generated sequences and the distribution of their corresponding MIC values are shown in Fig 2. Detailed information on the generation dataset is provided in Table 1.

**Table 1:** Dataset information of the generation task.

Sequence Length	Sample Size	Distribution of MIC Values				
		Minimum	25th Percentile	Median	75th Percentile	Maximum
4 ~ 9	664	-2.3274	0.4313	0.9247	1.4259	3.3979
10 ~ 19	2983	-3.6990	0.4099	0.9031	1.3979	3.3979
20 ~ 29	1863	-2.6021	0.4501	0.9031	1.3979	3.3979
30 ~ 39	632	-2.3974	0.4233	0.9031	1.4515	3.3979

### 3.2 Performance evaluation metrics

For the classification task of the discriminator, accuracy, recall, sensitivity, specificity, and F1 score are employed to evaluate the classification performance of the proposed model.


$$\begin{array}{c} \text{ACC}=\frac{\text{TP}+\text{TN}}{\text{TP}+\text{TN}+\text{FP}+\text{FN}} \end{array}$$ (14)

$$\begin{array}{c} \text{Precision}=\frac{\text{TP}}{\text{TP}+\text{FP}} \end{array}$$ (15)

$$\begin{array}{c} \text{Recall}=\frac{\text{TP}}{\text{TP}+\text{FN}} \end{array}$$ (16)

$$\begin{array}{c} \text{Specificity}=\frac{\text{TN}}{\text{TN}+\text{FP}} \end{array}$$ (17)

$$\begin{array}{c} \text{F1}=\frac{\text{2}\times \text{Precision}\times \text{Recall}}{\text{Precision}+\text{Recall}} \end{array}$$ (18)

TP represents the number of positive samples correctly predicted as positive, TN denotes the number of negative samples correctly predicted as negative, FP indicates the number of negative samples incorrectly predicted as positive, and FN signifies the number of positive samples incorrectly predicted as negative.

For regression tasks, mean squared error, root mean squared error, mean absolute error, and the R^2^ coefficient of determination are employed to evaluate the performance of predicting MIC values.


$$\begin{array}{c} \text{MSE}=\frac{\text{1}}{\text{m}}\sum_{\text{i}=\text{1}}^{\text{m}}{\left({\text{y}}_{\text{i}}-\hat{{\text{y}}_{\text{i}}}\right)}^{\text{2}} \end{array}$$ (19)

$$\begin{array}{c} \text{RMSE}=\sqrt{\frac{\text{1}}{\text{m}}\sum_{\text{i}=\text{1}}^{\text{m}}{\left({\text{y}}_{\text{i}}-\hat{{\text{y}}_{\text{i}}}\right)}^{\text{2}}} \end{array}$$ (20)

$$\begin{array}{c} \text{MAE}=\frac{\text{1}}{\text{m}}\sum_{\text{i}=\text{1}}^{\text{m}}\left|({\text{y}}_{\text{i}}-\hat{{\text{y}}_{\text{i}}})\right| \end{array}$$ (21)

$$\begin{array}{c} {\text{R}}^{\text{2}}=\text{1}-\frac{{\sum_{\text{i}=\text{1}}^{\text{m}}\left({\text{y}}_{\text{i}}-\hat{{\text{y}}_{\text{i}}}\right)}^{\text{2}}}{{\sum_{\text{i}=\text{1}}^{\text{m}}\left({\text{y}}_{\text{i}}-\overset{―}{\text{y}}\right)}^{\text{2}}} \end{array}$$ (22)

where ${\text{y}}_{\text{i}}$ is the ground truth MIC value of sample i, $\hat{{\text{y}}_{\text{i}}}$ is the corresponding predicted MIC, $\overset{―}{\text{y}}$ is the mean of the ground truth MIC value.

To perform the generation task, highly matched fragments were first retained through multiple sequence alignment and then combined with an MIC-guided sequence generation method to construct candidate AMP sequences. As sequence generation is constrained by conserved fragments in known AMPs, traditional evaluation metrics such as diversity and novelty are not sufficient to fully evaluate generation quality. In this study, predicted MIC was used as the optimization objective and selection criterion during generation, with lower values indicating better antimicrobial potency.

To provide a more comprehensive evaluation of generated sequences, we further considered both sequence-level and physicochemical metrics. At the sequence level, uniqueness [33] was defined as the proportion of non-duplicate sequences among all generated sequences; diversity was defined as the mean pairwise normalized Levenshtein distance within the generated set; edit-distance-based novelty was defined as the average normalized Levenshtein distance between each generated sequence and its nearest AMP in the training set; and similarity to known AMPs was defined as one minus this normalized nearest-neighbor distance. The normalized distance was computed as the Levenshtein distance divided by the maximum sequence length of the two sequences. These metrics were used to characterize redundancy, internal sequence variation, and distance from known AMP space, but were not treated as stand-alone optimization targets.

In addition, we selected a set of physicochemical parameters that were closely related to antimicrobial function as evaluation criteria. These were normalized hydrophobicity, normalized hydrophobic moment, net charge, isoelectric point, penetration depth, tilt angle, in vitro aggregation tendency, and amphiphilicity index. As these metrics were closely associated with AMP membrane disruption capacity and stability [17,34], the potential functionality of the sequence within biofilm environments provided a biologically meaningful basis for functional evaluation.

### 3.3 Classification results of the proposed model versus baseline models

To validate the superior performance of the proposed classifier in predicting AMP sequences, we compared it with multiple classifiers, including recently proposed models such as AMPlify [35], SBSM-Pro [36], and AMP-CLIP [12], and classical neural network models. Since our model integrates CNN and enhanced NCGCN feature extraction modules, the comparison with CNN also serves as an indirect ablation to validate the feature fusion strategy. The dataset was randomly partitioned five times into training, validation, and test sets at an 8:1:1 ratio. Experiments were conducted on a server with an NVIDIA RTX 4090 GPU using PyTorch 1.11.0 (CUDA 11.3). Table 2 lists the performance metrics of each model on the validation set. As the SBSM-Pro model only divides the dataset into training and test sets, it has not been included in Table 2. Table 3 presents the prediction performance of different models on their corresponding test sets. AMP-Hunter achieved the highest accuracy, recall, and F1 scores on both validation and test sets, which highlights its strong overall predictive power and particular suitability for AMP screening in scenarios where sensitivity is critical. Although the precision and specificity of AMP-Hunter were slightly lower than those of AMP-CLIP, its performance was more balanced across evaluation metrics, which reduced the risk of bias toward either positive or negative classes. The results show that the proposed model achieves the best performance across multiple metrics, which validates its effectiveness and superiority in the AMP prediction task.

**Table 2:** Performance of different models on the validation set for classification.

Model	ACC	Precision	Recall	Specificity	F1
AMP-Hunter	96.74 ± 0.37	97.52 ± 0.54	95.92 ± 0.74	97.56 ± 0.55	96.71 ± 0.37
AMPlify [35]	84.15 ± 0.74	78.69 ± 1.29	93.72 ± 1.20	74.57 ± 2.23	85.54 ± 0.54
AMP-CLIP [12]	93.48 ± 0.41	98.30 ± 0.59	88.46 ± 0.48	98.50 ± 0.54	93.12 ± 0.44
CNN	94.70 ± 0.50	96.82 ± 0.86	92.46 ± 0.35	96.94 ± 0.87	94.56 ± 0.49
Transformer	92.46 ± 0.29	92.68 ± 0.75	92.28 ± 0.52	92.70 ± 0.83	92.44 ± 0.25
LSTM	93.58 ± 0.40	95.72 ± 0.49	91.24 ± 0.51	95.88 ± 0.49	93.42 ± 0.38

**Table 3:** Performance of different models on the test set for classification.

Model	ACC	Precision	Recall	Specificity	F1
AMP-Hunter	95.82 ± 0.25	97.78 ± 0.58	93.90 ± 0.19	97.87 ± 0.58	95.80 ± 0.21
AMPlify [35]	84.48 ± 1.18	79.33 ± 1.30	93.29 ± 0.61	75.67 ± 1.80	85.74 ± 1.00
SBSM-Pro [36]	87.53 ± 1.01	89.24 ± 1.06	85.37 ± 1.67	89.70 ± 1.11	87.25 ± 1.09
AMP-CLIP [12]	93.54 ± 0.50	98.50 ± 0.25	88.40 ± 1.00	98.68 ± 0.24	93.20 ± 0.55
CNN	94.54 ± 0.32	97.16 ± 0.52	91.80 ± 0.73	97.34 ± 0.54	94.38 ± 0.34
Transformer	92.56 ± 0.25	92.78 ± 0.95	92.34 ± 1.31	92.82 ± 1.08	92.54 ± 0.31
LSTM	94.34 ± 0.50	95.50 ± 0.98	93.06 ± 0.55	95.58 ± 1.01	94.26 ± 0.48

### 3.4 Regression results of the proposed model versus baseline models

Furthermore, to evaluate the comprehensive performance of the proposed AMP-Hunter architecture in potent AMP prediction, its capability in predicting MIC values was additionally validated to assist in screening candidate peptides with strong antibacterial activity. In the regression task, the proposed model was compared against multiple models including representative models such as AMPredictor [37] and AMP-READ [12] which were recently used for AMP regression tasks, and classical neural network architectures. The experiments used the same settings as those of the classification task. Table 4 lists the regression performance metrics of each model on the validation set. Table 5 lists the prediction performance of each model on the test set. AMP-Hunter outperformed all baselines in MSE, MAE, and R^2^ with only a marginally higher RMSE than that of LSTM on the validation set, which indicates stable robustness and generalization. These results suggest that the integration of global and local feature extraction in AMP-Hunter has led to a highly comprehensive representation of peptide sequences, which enables reliable prediction and robust generalization capability of both antimicrobial activity and potency.

**Table 4:** Performance of different models on the validation set for regression.

Model	MSE	RMSE	R^2^	MAE
AMP-Hunter	0.1590 ± 0.0087	0.3986 ± 0.0111	0.9195 ± 0.0045	0.2311 ± 0.0084
AMPredictor [37]	0.3274 ± 0.0406	0.5713 ± 0.5713	0.8466 ± 0.0164	0.3075 ± 0.0314
AMP-READ [12]	0.2583 ± 0.0184	0.5079 ± 0.0184	0.8359 ± 0.0130	0.3695 ± 0.0121
CNN	0.1904 ± 0.0188	0.4358 ± 0.0211	0.9012 ± 0.0100	0.2474 ± 0.0085
Transformer	0.4524 ± 0.0296	0.6723 ± 0.0224	0.7362 ± 0.0134	0.4676 ± 0.0086
LSTM	0.3458 ± 0.0204	0.3770 ± 0.0235	0.6138 ± 0.0189	0.4412 ± 0.0094

**Table 5:** Performance of different models on the test set for regression.

Model	MSE	RMSE	R^2^	MAE
AMP-Hunter	0.1523 ± 0.0085	0.3901 ± 0.0109	0.9245 ± 0.0040	0.2305 ± 0.0081
AMPredictor [37]	0.3458 ± 0.0273	0.5876 ± 0.0253	0.8389 ± 0.0143	0.3272 ± 0.0262
AMP-READ [12]	0.2495 ± 0.0097	0.4994 ± 0.0097	0.8442 ± 0.0047	0.3639 ± 0.0056
CNN	0.1805 ± 0.0155	0.4245 ± 0.0182	0.9030 ± 0.0088	0.2487 ± 0.0065
Transformer	0.4408 ± 0.0137	0.6638 ± 0.0103	0.7365 ± 0.0111	0.4694 ± 0.0077
LSTM	0.3574 ± 0.0460	0.5969 ± 0.0373	0.7925 ± 0.0295	0.4340 ± 0.0271

### 3.5 Comparison with baselines models

To further evaluate the generation performance of AMP-Forge, we compared it with several representative peptide generation models, including Diffusion, GAN, LSTM, VAE, HydrAMP [17], ProGen [38], and AMPGen [25]. For fair comparison, each model was used to generate 5,000 peptide sequences, and the generated sets were evaluated using uniqueness, diversity, novelty, similarity to known AMPs, and predicted MIC. Among these metrics, predicted MIC was treated as the primary activity-oriented criterion, while the sequence-level metrics were used to assess redundancy, internal variation, and distance from known AMP space.

As shown in Table 6, AMP-Forge achieved the lowest predicted MIC among all compared models, indicating the strongest predicted antimicrobial potency. In addition, AMP-Forge maintained high uniqueness and high diversity, while preserving a moderate level of novelty and similarity to known AMPs. It should be noted that the relatively higher similarity of AMP-Forge to known AMPs, together with its correspondingly moderate novelty, is consistent with the architectural design of the model. Unlike fully unconstrained de novo generators, AMP-Forge first preserves conserved functional fragments through multiple sequence alignment and then performs local optimization in the unmatched regions. Therefore, AMP-Forge remains closer to the known AMP sequence space than generators that freely synthesize entire sequences. Despite this constraint, AMP-Forge still maintains high uniqueness and high diversity while achieving the best predicted MIC among the compared models, indicating that AMP-Forge generates sequences that are neither duplicates of known AMPs nor excessively distant from the known AMP sequence space, while still achieving favorable predicted potency. Overall, these results indicate that AMP-Forge provides a more favorable trade-off between potency-oriented optimization and sequence-level quality than the compared baseline generators. In particular, its advantage does not lie in maximizing novelty or diversity alone, but in generating candidate sequences with strong predicted activity while maintaining high uniqueness, substantial diversity, and a biologically reasonable distance from known AMP space.

**Table 6:** Comparison of AMP-Forge with representative peptide generators.

Model	Uniqueness	Diversity	Novelty	Similarity	Predicted MIC
AMP-Forge	0.9500	0.8280	0.3334	0.6666	0.8280
Diffusion	0.9566	0.7641	0.3621	0.6379	1.0636
GAN	0.8000	0.3736	0.6542	0.3458	1.2447
LSTM	0.8816	0.8346	0.3110	0.6890	1.3529
VAE	0.7350	0.7805	0.5142	0.4858	1.7516
HydrAMP [17]	0.9400	0.8647	0.585	0.4142	1.8238
ProGen [38]	0.8388	0.8151	0.2478	0.7522	1.2068
AMPGen [25]	0.8400	0.7964	0.4424	0.5576	1.3581

### 3.6 Ablation study of AMP-Hunter

To explicitly evaluate the contribution of each core component in AMP-Hunter, we conducted ablation experiments by removing or replacing key modules in the model. Specifically, this included removing the NCGCN branch and the CNN branch, using handcrafted sequence descriptors composed of amino acid composition, dipeptide composition, and physicochemical properties to replace ESM2-derived node representations; and using cosine similarity-based graph construction to replace PAM250-based graph construction strategy. The performance of these variants on the AMP classification and MIC regression tasks is presented in Tables 7–10.

**Table 7:** Ablation performance of AMP-Hunter on the validation set for classification.

Model	ACC	Precision	Recall	Specificity	F1
AMP-Hunter	96.74 ± 0.37	97.52 ± 0.54	95.92 ± 0.74	97.56 ± 0.55	96.71 ± 0.37
AMP-Hunter (without NCGCN)	94.70 ± 0.50	96.82 ± 0.86	92.46 ± 0.35	96.94 ± 0.87	94.56 ± 0.49
AMP-Hunter (without CNN)	87.68 ± 1.46	90.64 ± 1.73	84.09 ± 3.02	91.28 ± 1.86	87.21 ± 1.65
Handcrafted features instead of ESM2	90.88 ± 1.70	89.37 ± 2.93	92.99 ± 4.01	88.78 ± 3.89	91.06 ± 1.72
Cosine graph instead of PAM250 graph	91.49 ± 0.98	93.69 ± 6.07	89.94 ± 6.95	93.05 ± 7.15	91.32 ± 1.15

**Table 8:** Ablation performance of AMP-Hunter on the test set for classification.

Model	ACC	Precision	Recall	Specificity	F1
AMP-Hunter	95.82 ± 0.25	97.78 ± 0.58	93.90 ± 0.19	97.87 ± 0.58	95.80 ± 0.21
AMP-Hunter (without NCGCN)	94.54 ± 0.32	97.16 ± 0.52	91.80 ± 0.73	97.34 ± 0.54	94.38 ± 0.34
AMP-Hunter (without CNN)	86.25 ± 2.68	89.31 ± 3.87	82.50 ± 3.13	90.00 ± 4.13	85.72 ± 2.67
Handcrafted features instead of ESM2	90.34 ± 2.02	87.61 ± 3.30	94.15 ± 3.02	86.52 ± 4.29	90.70 ± 1.88
Cosine graph instead of PAM250 graph	91.16 ± 0.90	93.75 ± 5.76	89.03 ± 6.32	93.29 ± 6.60	90.94 ± 1.06

**Table 9:** Ablation performance of AMP-Hunter on the validation set for regression.

Model	MSE	RMSE	R^2^	MAE
AMP-Hunter	0.1590 ± 0.0087	0.3986 ± 0.0111	0.9195 ± 0.0045	0.2311 ± 0.0084
AMP-Hunter (without NCGCN)	0.1904 ± 0.0188	0.4358 ± 0.0211	0.9012 ± 0.0100	0.2474 ± 0.0085
AMP-Hunter (without CNN)	1.3993 ± 0.0234	1.1829 ± 0.0099	0.3449 ± 0.0151	0.9781 ± 0.0386
Handcrafted features instead of ESM2	0.9029 ± 0.1506	0.9476 ± 0.0787	-0.1255 ± 0.3632	0.8500 ± 0.0707
Cosine graph instead of PAM250 graph	0.9039 ± 0.1008	0.9492 ± 0.0545	0.1660 ± 0.1321	0.8411 ± 0.0491

**Table 10:** Ablation performance of AMP-Hunter on the test set for regression.

Model	MSE	RMSE	R^2^	MAE
AMP-Hunter	0.1523 ± 0.0085	0.3901 ± 0.0109	0.9245 ± 0.0040	0.2305 ± 0.0081
AMP-Hunter (without NCGCN)	0.1805 ± 0.0155	0.4245 ± 0.0182	0.9030 ± 0.0088	0.2487 ± 0.0065
AMP-Hunter (without CNN)	1.4176 ± 0.0506	1.1904 ± 0.0211	0.3453 ± 0.0262	0.9895 ± 0.0450
Handcrafted features instead of ESM2	0.7866 ± 0.1190	0.8848 ± 0.0684	0.2013 ± 0.2578	0.7983 ± 0.0664
Cosine graph instead of PAM250 graph	0.6963 ± 0.0675	0.8334 ± 0.0414	0.5456 ± 0.0656	0.7342 ± 0.0401

For AMP classification, the full AMP-Hunter model consistently achieved the best performance on both the validation and test sets. Removing the NCGCN module caused a consistent decrease in performance, with the test ACC and F1 declining from 95.82% and 95.80% to 94.54% and 94.38%, respectively. This indicates that graph-based relational learning provides complementary information beyond local sequence feature extraction.Removing the CNN branch caused the most substantial deterioration, reducing the test ACC and F1 to 86.25% and 85.72%, respectively, which highlights the critical role of local sequence pattern extraction in AMP identification. Replacing ESM2-derived node features with handcrafted sequence descriptors or replacing the PAM250-based graph with a cosine similarity-based graph also led to marked performance degradation, with test ACC values decreasing to 90.34% and 91.16%, respectively. These results confirm that both biologically informative node representations and evolution-aware graph topology contribute substantially to classification performance.

A similar trend was observed in the MIC regression task. The full AMP-Hunter model achieved the best overall performance, with a test MSE of 0.1523, RMSE of 0.3901, R^2^ of 0.9245, and MAE of 0.2305. Removing NCGCN reduced performance to a test MSE of 0.1805 and an R^2^ of 0.9030, suggesting that graph-based global feature propagation improves potency prediction. Removing the CNN branch caused the test MSE increasing to 1.4176 and the R^2^ dropping to 0.3453, further demonstrating the essential role of local sequence features in MIC estimation. Likewise, replacing ESM2-derived representations with handcrafted sequence descriptors or replacing PAM250-based graph construction with cosine similarity-based graph construction resulted in major performance losses, with test MSE values of 0.7866 and 0.6963, respectively. Overall, these ablation results show that the performance of AMP-Hunter does not arise from a single design choice alone. Although the CNN branch contributes the largest individual effect, NCGCN-based graph representation learning, ESM2-derived node features, and PAM250-guided graph construction each provide additional and consistent improvements. This indicates that the final performance of AMP-Hunter is achieved through the complementary integration of local sequence modeling and biologically informed graph-based representation learning.

### 3.7 Ablation study of AMP-Forge

To further evaluate the contribution of the major design components in AMP-Forge, we conducted ablation experiments by removing MIC guidance, Monte Carlo resampling, or MSA-based masking, respectively. We let he model variants with specific components removed and the complete AMP-Forge model each generate 5,000 sequences. As shown in Table 11, the full AMP-Forge achieved the best overall balance across these metrics, with the highest uniqueness, high diversity, moderate novelty, retained similarity to known AMPs, and the lowest predicted MIC. These results indicate that the complete generation strategy can simultaneously maintain sequence diversity, avoid excessive duplication, preserve AMP-related sequence characteristics, and optimize predicted potency.

**Table 11:** Ablation performance of AMP-Forge.

Model	Uniqueness	Diversity	Novelty	Similarity	Predicted MIC
AMP-Forge	0.9500	0.8280	0.3334	0.6666	0.8280
AMP-Forge (without MIC guidance)	0.6430	0.8207	0.2012	0.7988	1.0047
AMP-Forge (without Monte Carlo resampling)	0.5129	0.8219	0.1918	0.8082	1.1416
AMP-Forge (without MSA)	0.7000	0.787	0.5730	0.4270	1.1345

Removing MIC guidance led to a clear deterioration in predicted activity, uniqueness and novelty, while similarity to known AMPs increased. This suggests that without explicit MIC-oriented optimization, the generator tends to remain closer to known AMP patterns while losing part of its ability to explore more effective and non-redundant candidates. Removing Monte Carlo resampling caused the most severe reduction in uniqueness, and also led to the worst predicted MIC. Although the average diversity remained relatively high, the substantial drop in uniqueness indicates that the retained sequences became considerably more redundant. After removing the MSA mask, although the generated sequences exhibited the highest novelty, their diversity decreased, and the predicted MIC also increased significantly. This suggests that without aligning the sequences to preserve conserved regions, the generator would deviate further from known AMP sequence patterns; while this would increase apparent novelty, it would weaken the retention of biologically meaningful sequence features and reduce the ability to generate candidate molecules with well-predicted activity. Ablation experiment results indicate that AMP-Forge’s performance does not stem from a single component alone. Instead, MIC guidance, Monte Carlo resampling, and MSA-based masking play complementary roles. MIC guidance improves activity-oriented optimization, Monte Carlo resampling reduces redundancy and stabilizes candidate selection, and MSA-based masking preserves biologically meaningful sequence constraints during local optimization. The complete AMP-Forge model achieves the most balanced trade-off between predicting MIC, uniqueness, novelty, diversity, and the preservation of AMP-like features.

### 3.8 Feature distribution and sequence-level motif comparison of original sequences and generated sequences

**Fig 3:**
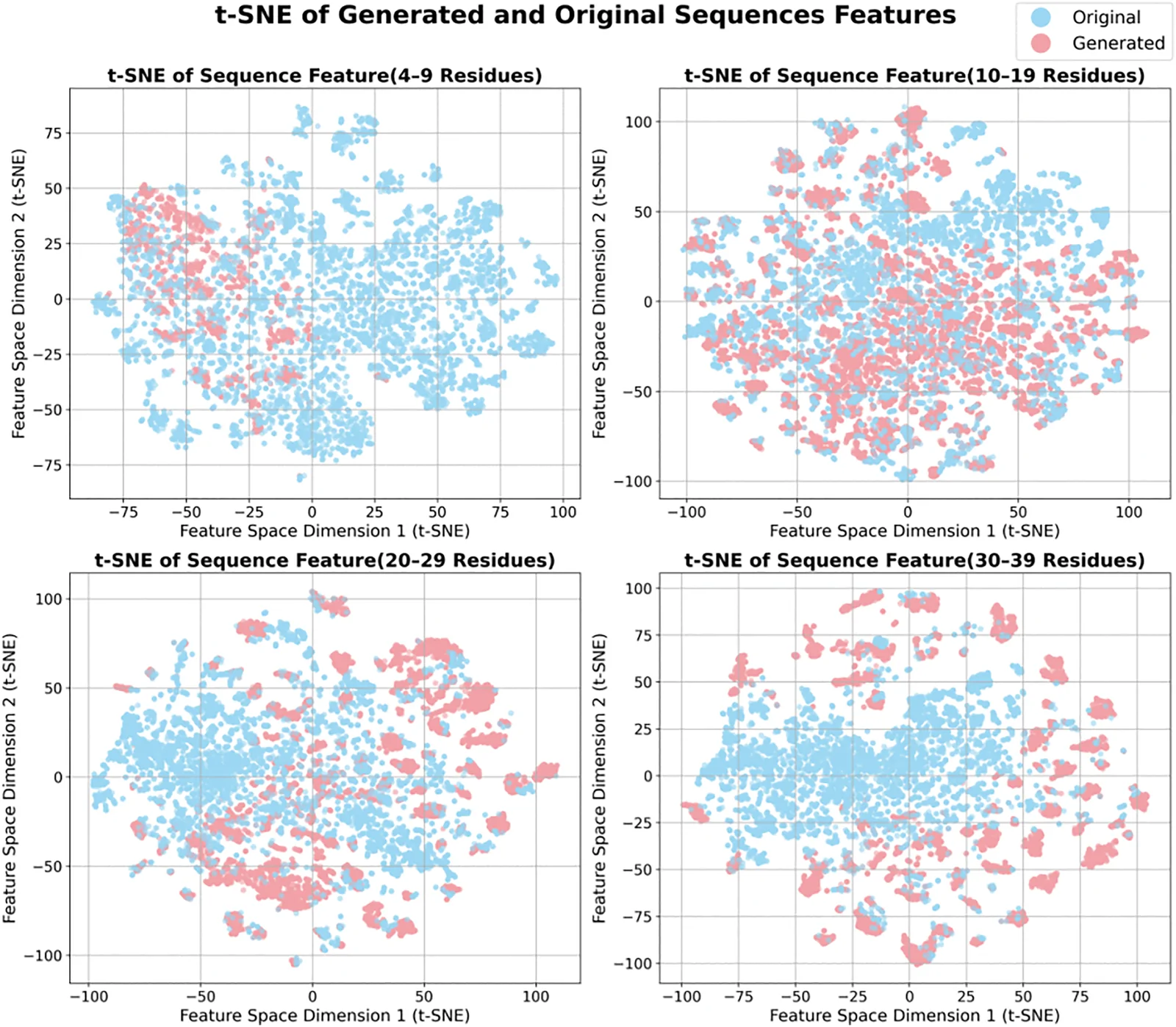
Feature distribution of original and generated sequences: ESM2 embeddings visualized by t-SNE showed that generated sequences overlapped with the original distribution, indicating successful feature space learning.

To analyze the distribution of the generated AMP sequences and original sequences in feature space, we used the large-scale protein language model ESM2 to extract features from both the original AMP dataset and the generated peptide sequences. This yielded a 1280-dimensional vector representation for each sequence. The generated peptide sequences were grouped into four categories based on the number of residues that they contained. For each group, the features of the optimized peptide sequences and those of the whole original dataset were visualized by reducing the high-dimensional representations to a two-dimensional space using t-SNE. Fig 3 shows the feature distribution results for the four groups and the original dataset. The visualization reveals that the sequences generated from different length groups showed a high degree of overlap with the original data in the feature space. As shown in Fig 3, the feature distribution of the generated peptide containing 4–9 residues fell within a subregion of the original peptide feature distribution. The feature distribution of the generated peptide containing 10–19 residues overlapped with the original peptide feature distribution within the same range, and the generated peptide sequence feature distribution filled areas that were not covered by the original peptide feature distribution. The generated peptide with 20–29 residues exhibited relatively uniform distribution at the center of the original peptide features. Although some generated sequences were distributed near the outer boundaries of the original sequence space, the overall feature distribution of the generated sequences largely remained consistent with that of the original sequences. Although the sequence features of generated peptides containing 30–39 residues were distributed in regions closer to the boundary of the original peptide feature range, they nevertheless showed high overlap with the original peptide feature distribution. This shows that the generated sequences were not randomly generated “noise;” rather, they were sequences with potential antimicrobial properties that were reasonably distributed within the original functional space.

To further validate whether the generated peptides retained biologically meaningful sequence characteristics beyond their distribution in feature space, we performed a sequence-level motif and residue-pattern comparison between the generated sequences and the original sequences within each peptide-length group. Because peptide length strongly affects residue composition and motif organization, the comparison was conducted against length-matched subsets of the original sequences dataset rather than against the full dataset.

**Fig 4:**
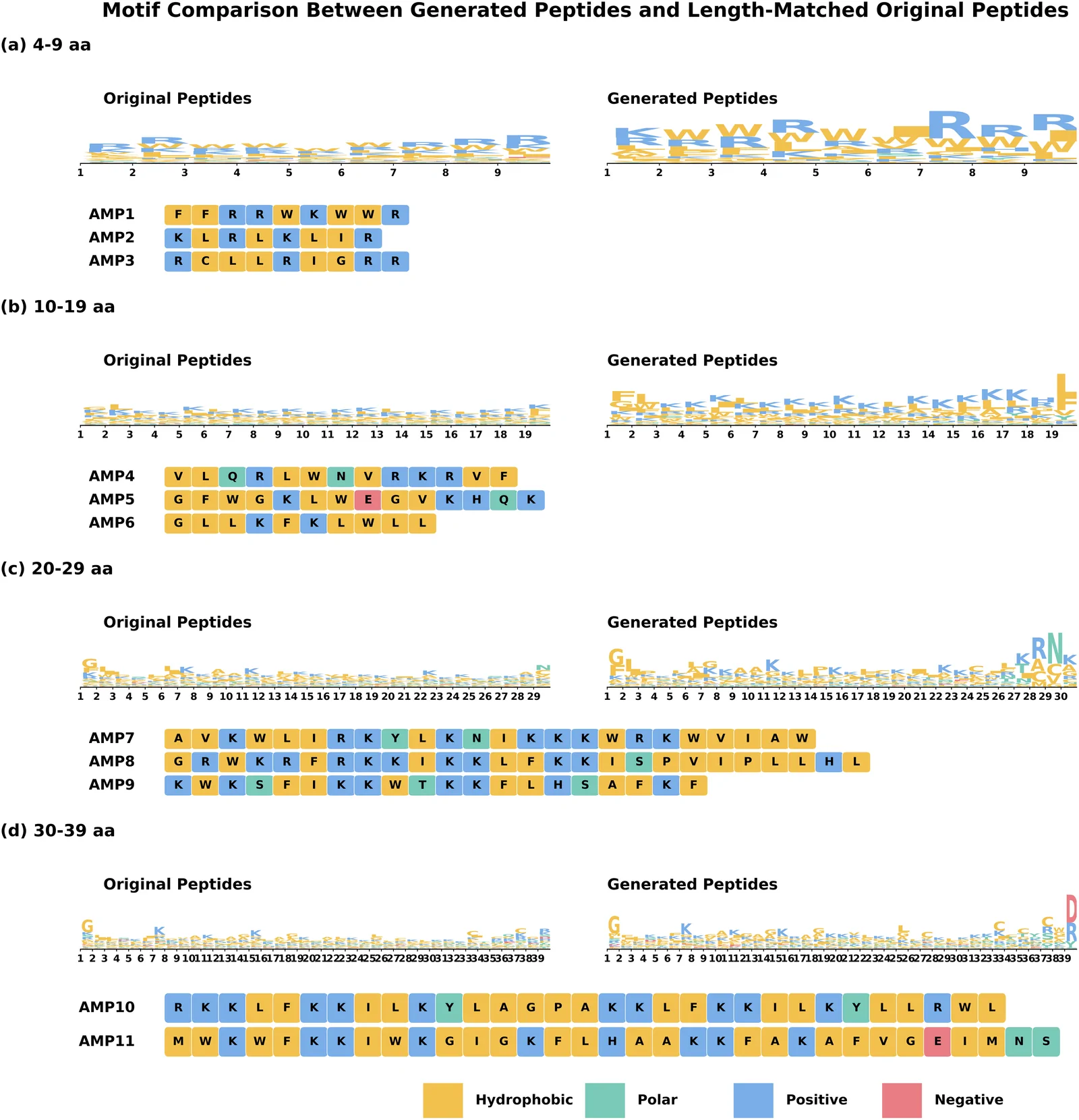
Sequence-level Motif Comparisons Between the Generated Peptides and the Original Peptides of Matching Lengths: (a) Motif comparison between the generated peptides and the original peptides in the 4–9 aa group. **(b)** Motif comparison between the generated peptides and the original peptides in the 10–19 aa group. **(c)** Motif comparison between the generated peptides and the original peptides in the 20–29 aa group. **(d)** Motif comparison between the generated peptides and the original peptides in the 30–39 aa group. The generated peptides consistently exhibit an enrichment of hydrophobic and positively charged residues, while showing differences from the original peptides at specific positions.

As shown in Fig 4, the generated peptides preserved several sequence-level characteristics including alternating distributions of hydrophobic and cationic residues, aromatic-basic combinations, and short cationic-rich segments which are commonly observed in the corresponding original peptides subsets. Across all four length groups, the generated peptides remained enriched in hydrophobic and positively charged residues, especially Lys, Arg, Trp, Leu, Ile, and Phe. This pattern is consistent with the canonical sequence architecture of many membrane-active AMPs, in which cationic residues facilitate electrostatic attraction to negatively charged bacterial membranes, whereas hydrophobic and aromatic residues promote membrane insertion and disruption. Notably, this enrichment pattern was not restricted to one specific length range, but was consistently observed from short peptides (4–9 residues) to longer peptides (30–39 residues), suggesting that AMP-Forge preserved broad antimicrobial sequence preferences during generation.

At the same time, the generated peptides are not simple replicas of the original sequences. The motifs in the generated peptide set differ from those in the original AMP subset in terms of specific preferences at multiple positions, indicating that the generation process introduces sequence variations while preserving biologically plausible residue usage patterns. This validates that AMP-Forge retains conserved sequence fragments and optimizes variable regions in the context of multiple sequence alignment.

### 3.9 Comparative analysis of physicochemical properties and predicted MIC values

**Fig 5:**
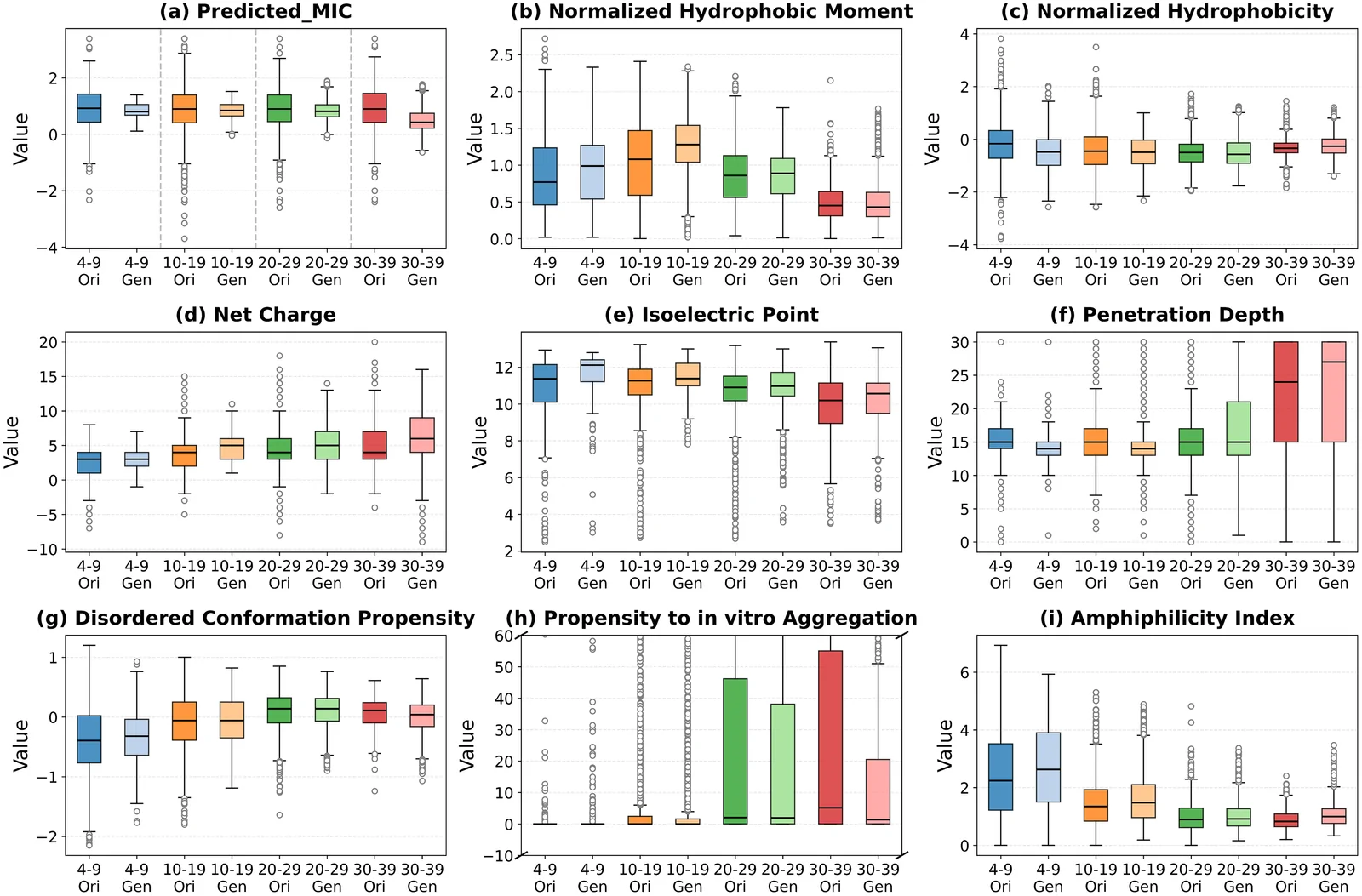
Comparison of MIC values and physicochemical properties between the original and generated sequences. **(a)** Predicted MIC **(b)** Normalized Hydrophobic Moment **(c)** Normalized Hydrophobicity **(d)** Net Charge **(e)** Isoelectric Point **(f)** Penetration Depth **(g)** Disordered Conformation Propensity **(h)** Propensity to in vitro Aggregation **(i)** Amphiphilicity Index.

In this study, 700 peptide sequences comprising 4–9 residues, 4,871 sequences comprising 10–19 residues, 5,658 sequences comprising 20–29 residues, and 5,826 sequences comprising 30–39 residues were retained. AMP-Hunter was then used to predict the MIC values of both the generated sequences and the original dataset sequences. Experimental results demonstrated that the predicted MIC values closely matched the actual values, validating the model’s high reliability. Table 12 lists the MIC values for the generated and original peptide sequences across different length intervals. Fig 5a presents boxplots of the MIC value distributions for both types of sequences. Combining the data in Table 12 and Fig 5a, we found that the generated peptide sequences exhibited lower predicted MIC values compared with those of the original sequences. Additionally, the MIC values of the generated sequences were highly concentrated, which indicates a more stable MIC range than those of the original sequences.

**Table 12:** MIC values for peptide sequences across different length intervals.

Sequence Source	4-9 Residues	10-19 Residues	20-29 Residues	30-39 Residues
Original Sequence	0.9102 ± 0.7822	0.8700 ± 0.8009	0.8859 ± 0.7623	0.7927 ± 0.7934
Generated Sequence	0.8617 ± 0.2610	0.8529 ± 0.2989	0.8476 ± 0.3353	0.5066 ± 0.4472

To evaluate the antimicrobial potential and stability of the generated sequences relative to the original ones, their physicochemical properties were compared. Given that generated sequences were locally optimized from original sequences within the same length intervals using latent space derived from multi-sequence alignment, targeted comparisons were conducted within corresponding length groups to avoid overly broad results. Table 13 presents the physicochemical properties of the generated peptide sequences across different length intervals. These were predicted using the DBAASP database according to Moon [39] et al.. Table 14 shows the corresponding physicochemical properties of the original peptide sequences for the same length intervals. Fig 5 presents the boxplots of the distribution of physicochemical properties between the generated sequence and the original sequence.

**Table 13:** Physicochemical properties of the generated sequences.

Physicochemical Properties	4-9 Residues	10-19 Residues	20-29 Residues	30-39 Residues
Normalized Hydrophobic Moment	0.946 ± 0.459	1.274 ± 0.395	0.862 ± 0.330	0.514 ± 0.320
Normalized Hydrophobicity	-0.461 ± 0.721	-0.468 ± 0.629	-0.502 ± 0.543	-0.246 ± 0.392
Net Charge	3.071 ± 1.408	4.692 ± 2.078	5.060 ± 3.017	6.073 ± 3.238
Isoelectric Point	11.631 ± 1.431	11.500 ± 0.831	10.823 ± 1.388	10.210 ± 1.464
Penetration Depth	14.207 ± 2.438	14.861 ± 3.653	17.145 ± 6.535	22.313 ± 8.548
Disordered Conformation Propensity	-0.352 ± 0.447	-0.064 ± 0.392	-0.094 ± 0.298	-0.009 ± 0.248
Propensity to in vitro Aggregation	23.704 ± 76.317	17.766 ± 72.718	59.389 ± 151.729	42.758 ± 117.552
Amphiphilicity Index	2.728 ± 1.423	1.623 ± 0.851	1.019 ± 0.477	1.057 ± 0.416

**Table 14:** Physicochemical properties of the original sequences.

Physicochemical Properties	4-9 Residues	10-19 Residues	20-29 Residues	30-39 Residues
Normalized Hydrophobic Moment	0.791 ± 0.427	1.136 ± 0.556	0.858 ± 0.400	0.522 ± 0.522
Normalized Hydrophobicity	-0.347 ± 0.875	-0.453 ± 0.772	-0.499 ± 0.526	-0.338 ± 0.338
Net Charge	2.173 ± 1.691	3.827 ± 2.084	4.253 ± 2.774	4.470 ± 4.470
Isoelectric Point	10.003 ± 2.936	11.034 ± 1.403	10.610 ± 1.621	9.847 ± 9.847
Penetration Depth	15.574 ± 5.310	15.456 ± 4.660	16.194 ± 6.378	21.866 ± 21.866
Disordered Conformation Propensity	-0.283 ± 0.553	-0.048 ± 0.429	-0.101 ± 0.318	-0.117 ± 0.117
Propensity to in vitro Aggregation	18.682 ± 81.804	21.667 ± 92.093	66.874 ± 158.609	46.890 ± 46.890
Amphiphilicity Index	2.458 ± 1.506	1.463 ± 0.789	1.001 ± 0.512	0.803 ± 0.803

The data presented in Tables 13–14 and Fig 5 show that the generated sequences exhibit higher net charge, higher isoelectric points, higher amphipathic index, and moderate penetration depths across all length groups compared with those of their original sequences. This indicates an increased tendency for the generated sequences to interact with negatively charged bacterial membranes [40]. In this study, MIC values were used as a fitness criterion to guide the directed evolution of peptide sequences with a focus on reducing MIC values during sequence evolution. However, focusing solely on fitness optimization may disrupt the regions of the candidate peptide sequences that are associated with antimicrobial properties. Unlike the methods that sacrifice physicochemical properties related to structural stability and antimicrobial performance, the proposed approach not only reduced MIC values from the original candidate sequences but also enhanced the physicochemical properties that were closely linked to antimicrobial efficacy in the generated sequences. Specifically, the generated sequences containing 4–9, 10–19, and 20–29 residues exhibited higher normalized hydrophobicity and lower normalized hydrophobic moment compared with those of the original sequences. This suggests that the generated sequences were less likely to cause cell lysis and that they possessed relatively high antimicrobial potential [41]. Moderately increased hydrophobicity enhances interactions with membrane hydrophobic regions, which enhances antimicrobial efficacy. Reduced hydrophobic moment may decrease non-specific binding to cell membranes, lower hemolytic risk and improve biosafety [42]. The generated sequences containing 10–19, 20–29, and 30–39 residues concurrently exhibited reduced propensity for in vitro aggregation. This suggests reduced toxicity to animal cells and low susceptibility to inactivation [43]. This characteristic effectively minimizes the likelihood of peptide formation into insoluble aggregates in physiological environments, resulting in decreased host cell toxicity and prolonged in vivo efficacy [44]. Furthermore, the generated sequences comprising 4–9 and 10–19 residues exhibited reduced disordered conformation propensity, which indicates that these peptides would readily form stable secondary structures in solution. This quality enhances structural stability and functional persistence of the peptide sequences within membrane environments [45]. A comparison of the generated and original sequences showed improvements in these physicochemical properties in the generated peptide sequences, which suggested improved antimicrobial potential and stability of the generated sequences. Notably, the optimization patterns of the generated peptides were not entirely uniform across length ranges. For peptides of 4–9 residues, the improvements were mainly characterized by increased net charge, elevated isoelectric point, and maintained amphiphilic index, suggesting that the optimization emphasized rapid electrostatic attraction to negatively charged bacterial membranes despite limited sequence length. For peptides of 10–19 residues, the generated sequences showed a more balanced improvement in charge, hydrophobicity, disorder propensity, and aggregation propensity, indicating optimization toward classical amphipathic AMP-like behavior with improved structural stability. For the 20–29 and 30–39 residue groups, the generated peptides exhibited stronger trends toward membrane-interaction-favorable profiles, including maintained amphiphilicity, increased charge, and physicochemical characteristics consistent with deeper membrane association. These observations suggest that AMP-Forge has learned different optimization preferences across various length ranges when generating sequences, corresponding to different underlying functional patterns, rather than a single, uniform sequence design pattern for all peptide lengths.

To evaluate membrane interaction potential, generated peptides were preliminarily screened by AMP-Hunter were screened using DBAASP [46], CAMP database [47] and AMP Scanner [48], followed by physicochemical filtering: normalized hydrophobicity moment > 0.5, normalized hydrophobicity within [-0.5, 0.5], net charge > +2, isoelectric point > 9.5, disordered conformation propensity < 0, propensity to in vitro aggregation = 0 and amphipathic index > 1.5 [49,50]. HELIQUEST [51] was used to further validate hydrophobicity and hydrophobic moment while analyzing the hydrophobic surface of the helical diagram to determine α-helix formation. DRAMP [52] database was applied to assess hemolytic activity. Structures of non-homologous peptides passing all filters were predicted using AlphaFold3 [53], yielding 11 candidates which were shown in Table 15 with their corresponding physicochemical properties across four length groups. Compared to the original sequences, the selected generated sequences avoid excessive hydrophobicity, which helps reduce non-specific toxicity, while exhibiting higher positive charge and a more favorable amphiphilic index. This facilitates binding to negatively charged bacterial membranes and enhances membrane-penetration potential. Notably, all generated peptides exhibited zero aggregation tendency, whereas the original dataset demonstrated greater instability and a stronger tendency to aggregate, indicating improved solubility and a reduced risk of aggregation.

**Table 15:** The filtered generated sequences.

No.	Sequence	Normalized Hydrophobic Moment	Normalized Hydrophobicity	Net Charge	Isoelectric Point	Penetration Depth	Disordered Conformation Propensity	Propensity to in vitro Aggregation	Amphiphilicity Index
AMP1	FFRRWKWWR	1	-0.35	4	12.41	15	-0.7	0	3.53
AMP2	KLRLKLIR	1.23	-0.14	4	12.15	14	-0.29	0	1.53
AMP3	RCLLRIGRR	1.19	-0.24	4	12.1	14	-0.33	0	1.09
AMP4	VLQRLWNVRKRVF	1.3	-0.45	4	12.41	15	-0.12	0	1.48
AMP5	GFWGKLWEGVKHQK	0.9	0.23	2	10.44	18	-0.21	0	2.06
AMP6	GLLKFKLWLL	0.57	-1.45	2	10.73	13	0.43	0	1.43
AMP7	AVKWLIRKYLKNIKKKWRKWVIAW	1.07	-0.06	9	11.73	15	-0.28	0	2.64
AMP8	GRWKRFRKKIKKLFKKISPVIPLLHL	1.01	-0.15	10	12.47	15	-0.27	0	1.59
AMP9	KWKSFIKKWTKKFLHSAFKF	1.4	0.18	7	11.45	15	-0.31	0	2.05
AMP10	RKKLFKKILKYLAGPAKKLFKKILKYLLRWL	1.4	-0.36	12	11.49	15	-0.19	0	1.89
AMP11	MWKWFKKIWKGIGKFLHAAKKFAKAFVGEIMNS	1.6	-0.32	7	11.15	15	0.01	0	1.6

### 3.10 Molecular dynamics simulation parameter settings

**Fig 6:**
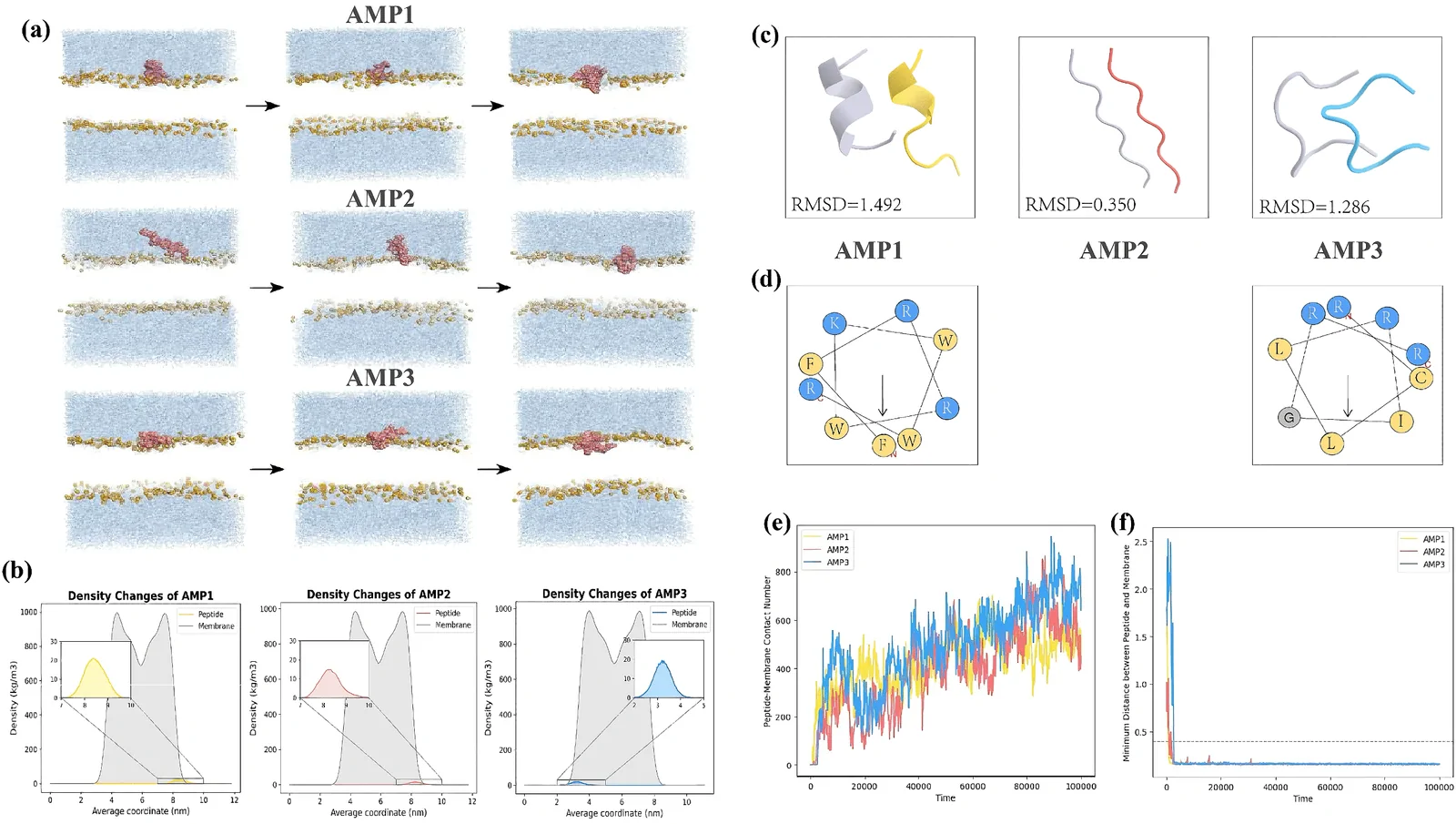
Molecular dynamics results for AMP1-AMP3. **(a)** Trajectory snapshots: Representative system snapshots during the 100 ns simulations showing peptide approach, membrane interaction, and insertion into the lipid bilayer. For visualization clarity, only peptides (pink), lipid head groups (yellow), and water molecules (blue) are displayed. **(b)** Density distribution: Density profiles of peptides and membranes along the membrane normal direction. Overlap between peptide and membrane densities indicates peptide insertion into the bilayer. **(c)** Structural alignment: Structural comparison between generated peptides (colored) and their corresponding parent sequences (gray) predicted by AlphaFold3, demonstrating strong structural conservation. **(d)** Helical wheel diagrams: Amphipathic α-helical projections of the generated peptides, illustrating spatial segregation of positively charged and hydrophobic residues. **(e)** Peptide-membrane contacts: Time evolution of the number of peptide-membrane contacts using a 0.4 nm cutoff, reflecting progressively strengthened peptide-membrane interactions. **(f)** Minimum distance: Minimum peptide-membrane distance during the 100 ns simulations. The dashed line indicates the 0.4 nm contact threshold corresponding to stable peptide-membrane association.

**Fig 7:**
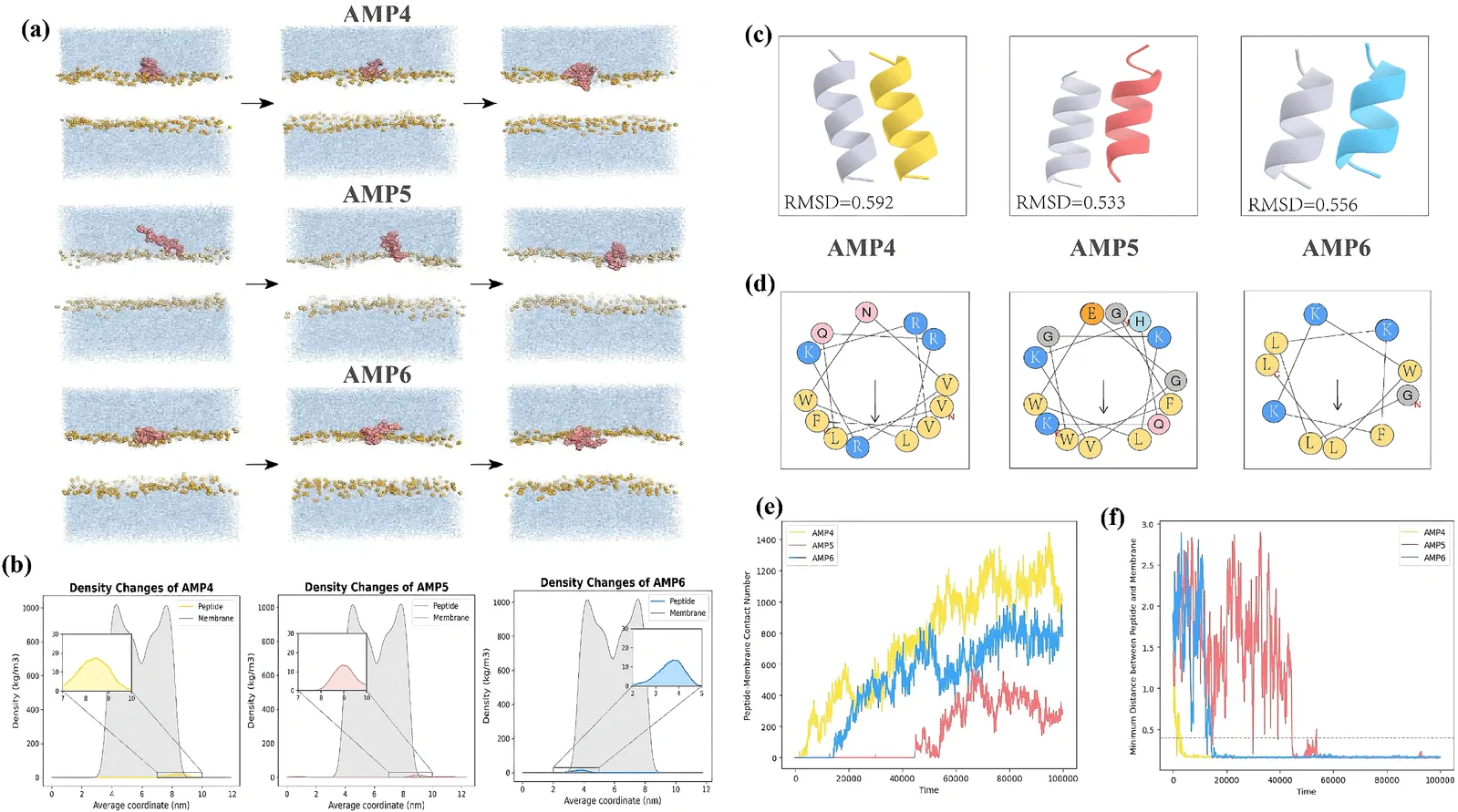
Molecular dynamics results for AMP4-AMP6. **(a)** Trajectory snapshots **(b)** Density distribution **(c)** Structural alignment **(d)** Helical wheel diagrams **(e)** Peptide-membrane contacts **(f)** Minimum distance.

**Fig 8:**
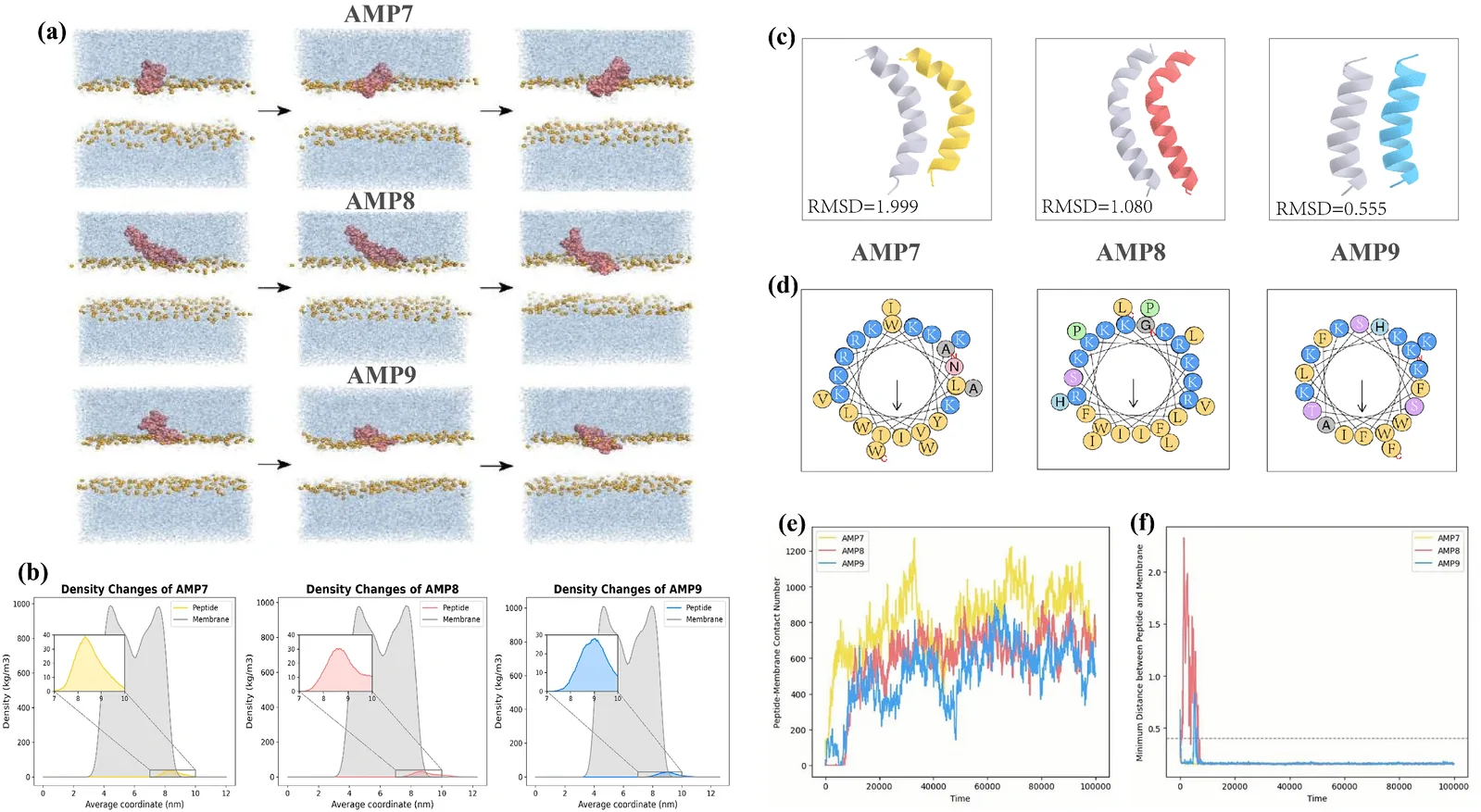
Molecular dynamics results for AMP7-AMP9. **(a)** Trajectory snapshots **(b)** Density distribution **(c)** Structural alignment **(d)** Helical wheel diagrams **(e)** Peptide-membrane contacts **(f)** Minimum distance.

**Fig 9:**
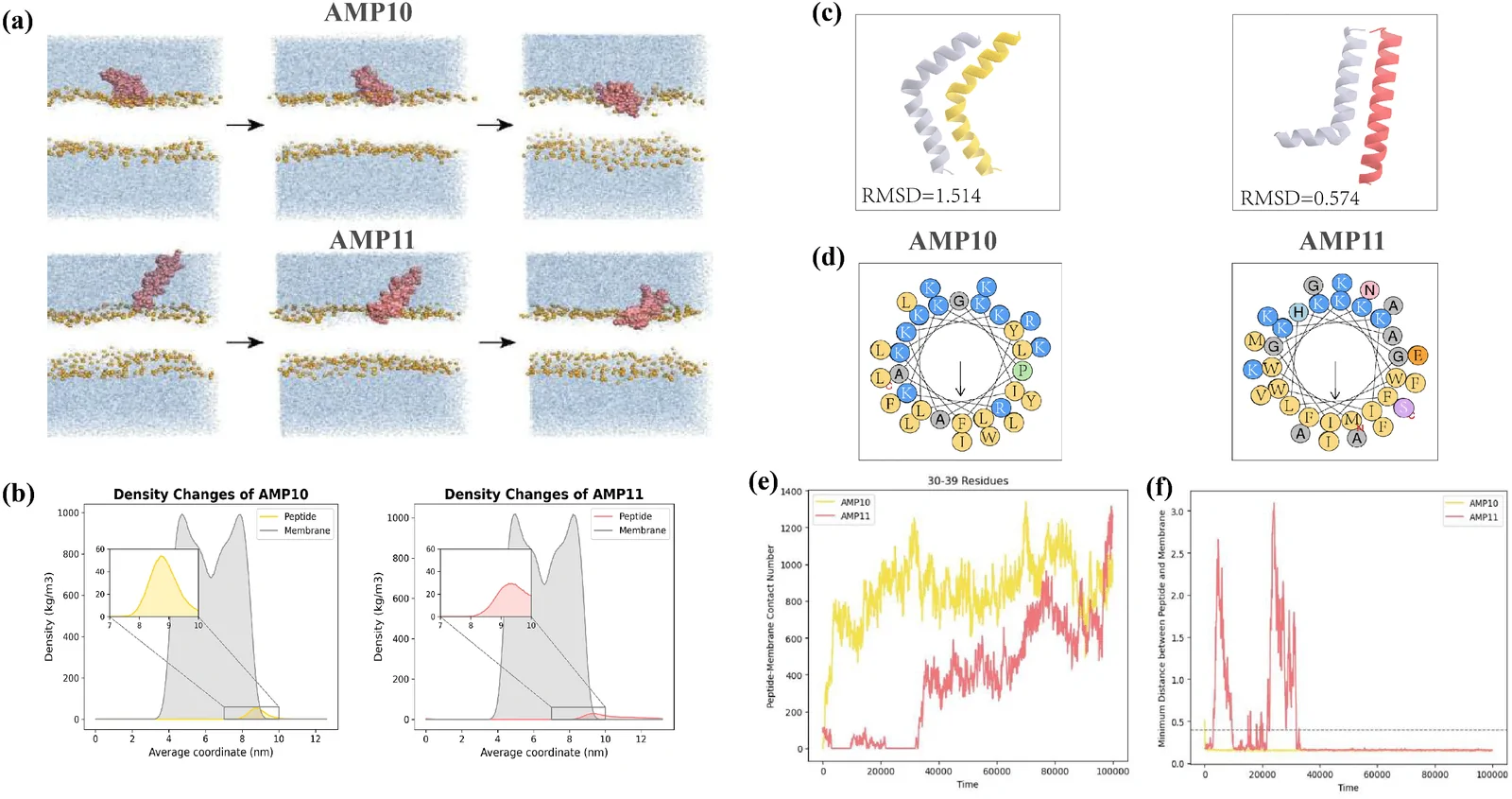
Molecular dynamics results for AMP10-AMP11. **(a)** Trajectory snapshots **(b)** Density distribution **(c)** Structural alignment **(d)** Helical wheel diagrams **(e)** Peptide-membrane contacts **(f)** Minimum distance.

Simulations were performed in the CHARMM36 force field [54]. A symmetric POPE/POPG (1:1) lipid bilayer was used to mimic the Gram-negative bacterial inner membrane, solvated with TIP3 water and 0.15 M KCl under physiological conditions (310 K, 1 bar, NPT ensemble). After energy minimization and equilibration with gradually released constraints, 100-ns production simulations were conducted. To analyze the active trajectories of the peptide sequences during the production simulation, three snapshots were captured during the total 100–ns timeframe to visualize the interaction states between the peptide and the membrane. The trajectory screenshot in Figs 6a, 7a, 8a and 9a shows the conformational changes in the screened peptide with varying numbers of residues relative to the lipid bilayer during the simulation. This depicts the dynamic process from the initial state to membrane insertion. To depict the trajectories of the peptides approaching and inserting into the membrane with improved clarity, only water molecules, head atoms of the bilayer, and peptide molecules were retained in the image.

### 3.11 Membrane interaction analysis of selected peptides

Furthermore, we elucidated the molecular-level mechanism of action for the generated AMPs by performing MD simulations. Prior sequence generation and screening results have shown that although the physicochemical properties, structural predictions, and the MICs predicted by the proposed models aided in inferring the potential antimicrobial performance, these data do not directly reveal the dynamic processes of peptide–membrane interactions. Hence, we simulated the real bacterial cell membrane environment through MD simulations. The generated peptide sequences were placed into corresponding systems under stimulated physiological conditions on a 100-ns timescale. The movement trajectories of the peptide and changes in the cell membrane showed the binding, insertion, and conformational changes that occur between the peptides and the cell membrane on the nanosecond timescale. This enabled an intuitive observation of key phenomena such as peptide adsorption on the membrane surface, induced membrane perturbations, and insertion into the membrane. This provides in silico insight of their membrane-binding capacity and stability. The 100-ns MD simulations are shown in Figs 6a, 7a, 8a and 9a. All 11 peptides exhibited a tendency to interact significantly with the lipid bilayer. In the initial phase, the peptides formed initial contact with the polar head groups of the membrane upon approach. As the simulation progressed, AMP1, AMP4, AMP7, and AMP10 exhibited pronounced insertion behavior, such as penetration into the polar head region and entering the hydrophobic tail region. This caused increased local membrane disruption, which led to loosened lipid packing and reduced density in the membrane midline region. Additionally, AMP2, AMP5, and AMP8 showed varying degrees of partial insertion into the membrane. Their extensive interactions with the membrane weakened its integrity and caused local disturbances on the membrane surface by affecting lipid alignment. Furthermore, AMP3, AMP6, AMP9, and AMP11 partially inserted into the hydrophobic layer of the membrane while another portion remains on the membrane surface. This was accompanied by strong membrane disruption effects, that significantly destabilized the bilayer structure and reduced the thickness of the membrane bilayer. The molecular dynamics results also suggested length-dependent differences in membrane interaction behavior. The shorter peptides were more likely to exhibit surface adsorption and shallow insertion, consistent with a mechanism dominated by charge-driven membrane association. In contrast, peptides in the intermediate and longer length ranges more frequently displayed sustained insertion, broader peptide-membrane contact interfaces, and more pronounced local membrane deformation, indicating that increased sequence length may enable a more stable segregation of hydrophobic and cationic regions and thereby support deeper membrane perturbation. These findings suggest that, while the generated peptides remain broadly consistent with classical membrane-targeting AMP behavior, the specific optimization pattern may vary with peptide length. Additionally, by calculating parameters such as the density distribution of peptides and membranes, contact numbers, and minimum distances, experimental predictions can be further supported by dynamic simulation data.

### 3.12 Density distribution of selected peptides and membranes

Figs 6b, 7b, 8b and 9b display the density distributions of peptides and membranes along the membrane normal direction. The overlapping distributions effectively indicate the contact and insertion status of the peptide with the membrane. The gray shaded region indicates the density distribution of the membrane, whereas the areas shaded in other colors correspond to the density distributions of the peptides from the different length groups. The membrane density distribution exhibits a concave depression between two peaks, which is a phenomenon that is associated with the structural characteristics of the lipid bilayer in simulations. This concave region corresponds to the hydrophobic core region of the lipid bilayer. The central depression corresponds to membrane thinning and disruption of the hydrophobic core, which further indicates interactions between the peptide and the membrane. Partial insertion of the peptide into the membrane causes local density perturbations and reduces membrane thickness in that region [55]. All peptides shown in Fig 5 interacted with the membrane and exhibited varying degrees of insertion. AMP1, AMP2, and AMP3 were primarily concentrated in the head-to-chain transition region of the membrane and showed shallow insertion near the membrane surface. The density distributions of AMP4, AMP5, and AMP6 showed overlapping density peaks on one side of the membrane with the peptide density peaks, which indicates that the peptides extended to the edge of the hydrophobic core and caused local membrane indentations. Notably, the density peak of AMP4 partially overlapped with the density distribution region of the outer lipid head group and chain of the membrane. This overlap was accompanied by the presence of the peptide binding interface near the central depression of the membrane, which caused a density depression in the membrane. Additionally, the MD simulation trajectory distinctly shows that the peptide–membrane interaction induced localized elastic deformation of the membrane. Peptides AMP7, AMP8, and AMP9 exhibited significantly deep insertion, and their density peaks coincided with the membrane midline, which indicates substantial overlap between peptide density and the hydrophobic core of the membrane. Among these, AMP9 insertion created the deepest membrane density trough, which caused the most pronounced local membrane thinning and thickness disturbance. Peptides containing 20–29 residues exhibited a broader density distribution range compared with that of those with < 20 residues. Their density persisted even at points where membrane density approached zero. This phenomenon is attributed to their greater residue count, and it indicates that the peptide sequences were not merely lying flat on the membrane surface but were partially inserted into the membrane in a relatively vertical orientation. The density distribution of the 30–39 residue peptide AMP10 overlapped with the core hydrophobic region of the membrane at 7.5–8.5nm, which indicates partial insertion into this area. Additionally, a locally deepened depression appeared at the membrane midline, where the peptide significantly disturbed the local thickness and chain orientation of the right leaflet. The density peak of the AMP11 peptide was closer to the outer edge of the right-side membrane peak and primarily overlapped with the head region. Its extension into the membrane and coverage of the centerline were minimal, but the density in the core region of the membrane decreased significantly, which indicates that the peptide bound to the membrane and disturbed it. Owing to limited simulation time, most peptides partially inserted into the membrane without traversing it, despite which they exhibited pronounced membrane disruption. Their interactions significantly altered membrane thickness, enabling interaction with the simulated bacterial membrane and supporting the predicted antimicrobial potential.

### 3.13 Generated peptide helical wheel diagram and structural comparison with parent peptide

The sequence generation method proposed in this study retained highly matched fragments after multiple sequence alignment. For unmatched regions, the proposed generator used directed evolution guided by MIC values in the latent space combined with resampling techniques to generate optimized sequences. Compared with that of their corresponding original sequences, the generated sequences not only retained antimicrobial activity but also exhibited lower MIC values, which enhanced their antimicrobial potential. Extensive studies have confirmed that AMP structure influences physicochemical properties such as amphiphilicity and hydrophobic moment [56,57]. These properties govern peptide–membrane interactions and ultimately indicate a close correlation between peptide structure and antimicrobial function. To validate structural similarity between the generated and corresponding original sequences and confirm the inheritance of antimicrobial activity, we used AlphaFold3 to generate structures. We then aligned each selected generated peptide with its original counterpart.. Peptide chain structures predicted using AlphaFold2 provide substantial support for structure-aware machine learning classifiers and the interpretation of peptide activity mechanisms [58]. Therefore, using AlphaFolds3 to generate peptide sequence structures for comparison is reliable.

Figs 6c, 7c, 8c and 9c show that the structure of the generated peptide sequence predicted using AlphaFold3 exhibited high consistency with its corresponding original peptide sequence. Across the four length categories, the RMSD values calculated from structural alignment were generally small, with most paired backbone RMSDs being low. Typically, RMSD was ≤1.5 Å, with only a few approaching 2.0 Å. In most cases, the major chain positions and helical geometries were preserved, which indicates structural conservation between the generated and original sequences. Given that the geometry of amphipathic helices, hydrophobic moment, and the spatial positioning of charged residues are key determinants of membrane binding and disruption behavior, the structural conservation of the generated sequences supports their retention of the antimicrobial potential of the original peptide sequences. Regarding confidence metrics, all regions of the 11 peptide sequences exhibited pLDDT values > 70%. At the local conformation level, the generated sequences maintained highly consistent folding patterns with the original sequences, with the vast majority of regions > 90%. The predicted reliability of the main chain was notably high, which supports our structural similarity inference based on RMSD. Regarding another confidence metric, pTM, peptides shorter than 20 residues are often unstable and of limited significance owing to their lack of complex global topology. For the peptides focused on in this study, pLDDT was generally more reliable than pTM [53].

We further validated the hydrophobicity and hydrophobic moment of the generated peptide using HELIQUEST. Combined with the helical wheel diagrams, this analysis determined whether the peptide sequences formed amphipathic α-helices. Figs 6d, 7d, 8d and 9d shows the helical diagrams for different peptide sequences except for the AMP2 sequence, which is shorter than 8 residues and thus unable to generate a helical diagram. All generated peptides exhibited typical amphipathic α-helix characteristics such as the concentration of positively charged residues on one side of the helix, while hydrophobic residues formed a relatively continuous hydrophobic surface. This spatial segregation is conducive for the peptide to adsorb onto the negatively charged bacterial membrane surface via electrostatic interactions and insert into the hydrophobic regions of the membrane, thereby disrupting the membrane structure. The aromatic residues present in some peptides further enhance the insertion force and membrane disruption capability; however, they may simultaneously increase non-specific interactions with mammalian membranes. Another group of peptides containing polar and neutral residues increases flexibility and enhances selectivity. This indicates gentler interaction on mammalian cells.

Different peptides exhibited diversity in the composition and distribution of hydrophilic and hydrophobic residues, which determines variations in their modes of action. In Fig 9, the blue residues represent positively charged residues, and are concentrated on one side of the helix, whereas the yellow residues on the opposite side are hydrophobic residues that form a strong hydrophobic surface that separates from the hydrophilic surface. AMP1, AMP4, AMP7, and AMP10 possessed strong hydrophobic surfaces with clustered aromatic residues and exhibited high insertion force capable of inducing significant membrane disruption [50]. However, the aggregation of positive charges and aromatic residues may cause non-specific damage to mammalian cell membranes. AMP3, AMP8, AMP9, and AMP11 accumulated positive charges on their hydrophilic surfaces and exhibited strong adsorption to bacterial membranes, which is particularly effective against negatively charged Gram-negative bacteria. The introduction of residues such as glutamic, glycine, or proline enhances solubility and flexibility while maintaining membrane-binding capacity and preventing aggregation caused by excessive rigidity. This approach increases the potential for selective antimicrobial competence while reducing toxicity and aggregation risks [59]. AMP5 and AMP6 maintained overall amphiphilicity and incorporated negatively charged or neutral residues. Although the presence of negative residues may weaken the initial electrostatic attraction between the peptide and bacterial membranes, the overall sequence remains positively charged, which minimizes the impact. Furthermore, the presence of negative residues partially mitigates the risk of non-specific hemolysis [34]. Overall, the generated peptides exhibited both effective membrane-binding potential and diverse regulatory mechanisms, which indicates promising antimicrobial activity.

### 3.14 Contacts number and minimum distance between generated peptides and membrane

Figs 6e, 7e, 8e and 9e displays the number of contacts between peptides containing a varying number of residues and membranes at a contact threshold of 0.4 nm. This contact distance accurately reflects stable physical contact between the two entities at the van der Waals interaction level. Additionally, multiple simulation studies [60] used 0.4 nm as the distance threshold for counting contacts between peptides and lipid headgroups, which is a suitable metric for binding and insertion behavior. Moreover, other studies [61] used a distance threshold of ≥0.6 nm to count peptide–membrane contacts. Although atomic interactions may exist at this distance, it often merely indicates proximity to the membrane rather than stable contact. The 0.4-nm contact criterion used in this study is stricter than the commonly used 0.6-nm threshold, because it recognizes contact only when peptides and membrane atoms are within the van der Waals interaction range. Thus, it reflects substantive binding and insertion processes with improved clarity. Figs 6f, 7f, 8f and 9f shows the minimum distance variation between the peptide and the membrane. The stabilizing of the minimum distance within 0.4 nm indicates that the peptide is tightly bound to the membrane and exerts a stable effect.

The number of contacts between the generated peptides and the membrane during the simulation gradually increased and eventually stabilized; furthermore, it was accompanied by a decrease in the minimum distance that ultimately stabilized within 0.4 nm. This indicates that the peptides completed the process from surface adsorption to stable binding within a relatively short time. Within each length group, peptides such as AMP2, AMP4, AMP7, and AMP10 exhibited rapid and sustained contact growth through tightly binding and deep insertion into the membrane, which caused significant structural disruption. In contrast, the peptides AMP1, AMP3, AMP6, AMP8, and AMP9 exhibited a gradual insertion pattern, that was characterized by a steady increase in contact numbers, followed by stabilization. This suggests that these peptides established stable interactions with the membrane upon approaching its surface, and it indicates moderate membrane penetration capabilities. A few peptides such as AMP5 and AMP11 exhibited dynamic characteristics with large fluctuations in contact numbers and unstable minimum distances, which indicate highly dynamic interactions and intermittent binding with the membrane. This diversity is indicative of the varied modes of membrane action among synthetic peptides. For example, some insert deeply to disrupt hydrophobic core regions and enhance antimicrobial efficacy, whereas others bind dynamically to disturb the surface and weaken membrane stability. Collectively, these findings show that the generated peptides not only bind effectively to membranes but also use flexible and diverse modes of action, which is consistent with their potential antimicrobial mechanisms.

## 4 Discussion

In this study, we have proposed an integrated framework comprising a discriminator AMP-Hunter and a generator AMP-Forge for AMP recognition and generation. The AMP-Hunter uses the pre-trained protein language model ESM2 to extract sequence features as node features and combines the PAM250 evolutionary matrix to obtain peptide sequence similarity as edge relations. A graph neural network module was constructed based on node and edge information, and integrated with a convolutional neural network module to fully incorporate local features and global graph structure characteristics. This enhances the generalization and robustness of the model and enables effective classification of candidate sequences and accurate MIC prediction. Potent prediction and MIC prediction were achieved without altering the model architecture. Experimental results showed superior performance compared with those of multiple baseline models. The AMP-Forge processes within the latent space of a variational autoencoder guided by MIC values. It performs multiple sequence alignment on input sequences to retain high-matching regions associated with antimicrobial properties. By integrating resampling strategies, it continuously optimizes unmatched regions in the latent space and yields peptide sequences with physicochemical properties linked to superior antimicrobial potential and stability. The predictive accuracy of the discriminator provides reliable support for the generator and enables a seamless closed-loop workflow from discovery to optimization, prediction, and validation. This integrated approach significantly enhances the efficiency of identifying and generating high-potential AMPs. The generation strategy employed by AMP-Forge is an optimization approach that preserves conservatism. By retaining conserved fragments identified through multiple sequences alignment and introducing local sequence evolution based on minimum inhibitory concentration values in non-conserved regions, the strategy optimizes predicted MIC values without unduly compromising AMP-related structures and physicochemical properties. This framework is suitable for enhancing the activity of antimicrobial peptides while maintaining biological plausibility and reducing the instability typically associated with fully random generation. The results indicate that the generated peptides still exhibit significant overlap with the distribution of the original AMPs in the ESM2 feature space, retaining sequence-level motif characteristics while demonstrating improved predicted MIC values and favorable physicochemical properties. However, this generation method may have a relatively limited scope for exploring novel patterns. To validate this framework, representative sequences across length categories were screened using the proposed discriminator, followed by multi-level filtering through embedded predictors in multiple databases and physicochemical property assessments. Eleven candidate sequences were selected for MD simulation evaluation. Visualized trajectories from MD simulations were combined with density distribution of peptide–membrane interactions, contact number variations, and minimum distance changes revealed that the generated peptides interact with bacterial membranes through stable adsorption, partial insertion, and disruption of bilayer integrity. These findings not only provide additional in silico support for the predicted antimicrobial potential of target sequences but also demonstrate the capability of the framework to design peptides with both superior activity and selectivity.

Despite the encouraging results, the current framework also has some limitations. First, both the AMP-Hunter and AMP-Forge were developed using relatively limited datasets, consisting primarily of short linear peptides and minimum inhibitory concentration annotations centered on E. coli. As a result, the generalizability of AMP-Hunter and AMP-Forge to antimicrobial peptides targeting other bacterial species or broader peptide types remains to be further evaluated. Second, the current workflow is primarily computational. The improved activity, selectivity, and safety of the generated peptides were inferred from predicted MIC values, physicochemical property, and molecular dynamics simulations rather than direct experimental measurements. Therefore, although these results support the promise of the proposed framework, further validation through antimicrobial activity assays, hemolysis tests, and cytotoxicity tests will be important to confirm the translational potential of the candidate peptides. And molecular dynamics validation has been conducted over limited simulation time scales, using a limited set of candidate molecules; thus, it may cannot fully capture the complexity of biological membranes or in vivo environments.

Overall, this study demonstrates that combining deep learning with physicochemical evaluations and relevant simulations can accelerate the computational discovery of novel antimicrobial peptides. Within the scope of the current dataset and validation setup, the optimized peptides retain the key structural features of their parent peptides while exhibiting enhanced predicted antimicrobial potential. Therefore, by integrating sequence prediction, sequence generation, and simulation validation, this method provides a practical and versatile framework for the prediction and design of antimicrobial peptides, offering support in addressing the challenge of antimicrobial resistance. Future work will involve further experimental validation to establish its translational value.

## Supporting information

S1 Table Sensitivity analysis of graph-construction threshold τ for AMP-Hunter classification on the validation set. This table presents the classification performance of AMP-Hunter on the validation set under different PAM250-based graph-construction thresholds (τ = 0.1–0.9), including accuracy, precision, recall, specificity, and F1 score.
(DOCX)

S2 Table Sensitivity analysis of graph-construction threshold τ for AMP-Hunter classification on the test set. This table presents the classification performance of AMP-Hunter on the test set under different PAM250-based graph-construction thresholds (τ = 0.1–0.9), including accuracy, precision, recall, specificity, and F1 score.
(DOCX)

S3 Table Sensitivity analysis of graph-construction threshold τ for AMP-Hunter regression on the validation set. This table presents the MIC regression performance of AMP-Hunter on the validation set under different PAM250-based graph-construction thresholds (τ = 0.1–0.9), including mean squared error (MSE), root mean squared error (RMSE), coefficient of determination (R^2^), and mean absolute error (MAE).
(DOCX)

S4 Table Sensitivity analysis of graph-construction threshold τ for AMP-Hunter regression on the test set. This table presents the MIC regression performance of AMP-Hunter on the test set under different PAM250-based graph-construction thresholds (τ = 0.1–0.9), including mean squared error (MSE), root mean squared error (RMSE), coefficient of determination (R^2^), and mean absolute error (MAE).
(DOCX)
