# Supplementary material for: A unified framework for potency-oriented AMP discovery via multi-modal learning and guided sequence synthesis
Source: PLoS Comput Biol. 2026 Sep 3;22(9):e1014771. doi: 10.1371/journal.pcbi.1014771 (PMC13568523; doi:10.1371/journal.pcbi.1014771)
Supplement: Supplementary file 1 [file pcbi.1014771.s001.docx]

**Appendix Table S1**

**Table S1. Performance of Different 𝜏 on the Validation Set for Classification**

| 𝜏 | ACC | Precision | Recall | Specificity | F1 |
| --- | --- | --- | --- | --- | --- |
| 0.1 | 95.43 | 97.15 | 93.60 | 97.26 | 95.34 |
| 0.2 | 96.65 | 98.11 | 95.12 | 98.17 | 96.59 |
| 0.3 | 95.58 | 97.46 | 93.60 | 97.56 | 95.49 |
| 0.4 | 95.43 | 96.86 | 93.90 | 96.95 | 95.36 |
| 0.5 | 95.73 | 97.47 | 93.90 | 97.56 | 95.65 |
| 0.6 | 95.73 | 97.47 | 93.90 | 97.56 | 95.65 |
| 0.7 | 95.73 | 97.47 | 93.90 | 97.56 | 95.65 |
| 0.8 | 95.58 | 97.16 | 93.90 | 97.26 | 95.50 |
| 0.9 | 95.73 | 97.47 | 93.90 | 97.56 | 95.65 |

**S1 Table. Sensitivity analysis of graph-construction threshold τ for AMP-Hunter classification on the validation set.**

This table presents the classification performance of AMP-Hunter on the validation set under different PAM250-based graph-construction thresholds (τ = 0.1–0.9), including accuracy, precision, recall, specificity, and F1 score.
