# Supplementary material for: A unified framework for potency-oriented AMP discovery via multi-modal learning and guided sequence synthesis
Source: PLoS Comput Biol. 2026 Sep 3;22(9):e1014771. doi: 10.1371/journal.pcbi.1014771 (PMC13568523; doi:10.1371/journal.pcbi.1014771)
Supplement: Supplementary file 2 [file pcbi.1014771.s002.docx]

**Appendix Table S2**

**Table S2. Performance of Different 𝜏 on the Test Set for Classification**

| 𝜏 | ACC | Precision | Recall | Specificity | F1 |
| --- | --- | --- | --- | --- | --- |
| 0.1 | 91.92 | 94.79 | 88.72 | 95.12 | 91.65 |
| 0.2 | 95.88 | 97.78 | 93.90 | 97.87 | 95.80 |
| 0.3 | 92.23 | 94.82 | 89.33 | 95.12 | 91.99 |
| 0.4 | 93.14 | 95.50 | 90.55 | 95.73 | 92.96 |
| 0.5 | 92.07 | 94.81 | 89.02 | 95.12 | 91.82 |
| 0.6 | 92.07 | 94.81 | 89.02 | 95.12 | 91.82 |
| 0.7 | 92.07 | 94.81 | 89.02 | 95.12 | 91.82 |
| 0.8 | 92.07 | 94.81 | 89.02 | 95.12 | 91.82 |
| 0.9 | 92.07 | 94.81 | 89.02 | 95.12 | 91.82 |

**S2 Table. Sensitivity analysis of graph-construction threshold τ for AMP-Hunter classification on the test set.**

This table presents the classification performance of AMP-Hunter on the test set under different PAM250-based graph-construction thresholds (τ = 0.1–0.9), including accuracy, precision, recall, specificity, and F1 score.
