# Supplementary material for: A unified framework for potency-oriented AMP discovery via multi-modal learning and guided sequence synthesis
Source: PLoS Comput Biol. 2026 Sep 3;22(9):e1014771. doi: 10.1371/journal.pcbi.1014771 (PMC13568523; doi:10.1371/journal.pcbi.1014771)
Supplement: Supplementary file 3 [file pcbi.1014771.s003.docx]

**Appendix Table S3**

**Table S3. Performance of Different 𝜏 on the Validation Set for Regression**

| 𝜏 | MSE | RSME | R2 | MAE |
| --- | --- | --- | --- | --- |
| 0.1 | 0.1626 | 0.4032 | 0.9187 | 0.2402 |
| 0.2 | 0.1586 | 0.3982 | 0.9207 | 0.2329 |
| 0.3 | 0.1603 | 0.4004 | 0.9198 | 0.2395 |
| 0.4 | 0.1594 | 0.3993 | 0.9200 | 0.2343 |
| 0.5 | 0.1595 | 0.3994 | 0.9205 | 0.2346 |
| 0.6 | 0.1616 | 0.402 | 0.9192 | 0.2462 |
| 0.7 | 0.1593 | 0.3991 | 0.9203 | 0.2328 |
| 0.8 | 0.1593 | 0.3991 | 0.9203 | 0.2328 |
| 0.9 | 0.1643 | 0.4053 | 0.9183 | 0.2513 |

**S3 Table. Sensitivity analysis of graph-construction threshold τ for AMP-Hunter regression on the validation set.**

This table presents the MIC regression performance of AMP-Hunter on the validation set under different PAM250-based graph-construction thresholds (τ = 0.1–0.9), including mean squared error (MSE), root mean squared error (RMSE), coefficient of determination (R²), and mean absolute error (MAE).
