# Supplementary material for: A unified framework for potency-oriented AMP discovery via multi-modal learning and guided sequence synthesis
Source: PLoS Comput Biol. 2026 Sep 3;22(9):e1014771. doi: 10.1371/journal.pcbi.1014771 (PMC13568523; doi:10.1371/journal.pcbi.1014771)
Supplement: Supplementary file 4 [file pcbi.1014771.s004.docx]

**Appendix Table S4**

**Table S4. Performance of Different 𝜏 on the Test Set for Regression**

| 𝜏 | MSE | RSME | R2 | MAE |
| --- | --- | --- | --- | --- |
| 0.1 | 0.1664 | 0.4079 | 0.9188 | 0.2471 |
| 0.2 | 0.1648 | 0.4060 | 0.9210 | 0.2386 |
| 0.3 | 0.1633 | 0.4041 | 0.9218 | 0.2401 |
| 0.4 | 0.1646 | 0.4057 | 0.9207 | 0.2385 |
| 0.5 | 0.1654 | 0.4066 | 0.9208 | 0.2381 |
| 0.6 | 0.1669 | 0.4085 | 0.9182 | 0.2485 |
| 0.7 | 0.1664 | 0.4079 | 0.9193 | 0.2386 |
| 0.8 | 0.1664 | 0.4079 | 0.9193 | 0.2386 |
| 0.9 | 0.1670 | 0.4086 | 0.9186 | 0.2479 |

**S4 Table. Sensitivity analysis of graph-construction threshold τ for AMP-Hunter regression on the test set.**

This table presents the MIC regression performance of AMP-Hunter on the test set under different PAM250-based graph-construction thresholds (τ = 0.1–0.9), including mean squared error (MSE), root mean squared error (RMSE), coefficient of determination (R²), and mean absolute error (MAE).
