# Supplementary material for: A unified framework for potency-oriented AMP discovery via multi-modal learning and guided sequence synthesis
Source: PLoS Comput Biol. 2026 Sep 3;22(9):e1014771. doi: 10.1371/journal.pcbi.1014771 (PMC13568523; doi:10.1371/journal.pcbi.1014771)
Supplement: Supplementary file 5 [file pcbi.1014771.s006.docx]

Response to Reviews

Manuscript ID: PCOMPBIOL-D-26-00846

Title: A Unified Framework for Potency-Oriented AMP Discovery via Multi-Modal Learning and Guided Sequence Synthesis

Dear Editors and Reviewers,

We sincerely thank you for your careful evaluation of our manuscript and for the constructive comments and suggestions. We have revised the manuscript thoroughly in response to all comments from the editors and reviewers. These revisions have helped us improve the clarity, rigor, reproducibility, and overall presentation of the work.

In the revised manuscript, we have clarified the methodological description, corrected inconsistencies in the text and algorithms, strengthened the discussion of related work and study limitations, added new ablation and benchmarking experiments, expanded the biological interpretability analysis of the generated peptides, and revised the wording of claims related to molecular dynamics simulations to more clearly distinguish computational evidence from experimental validation. We have also carefully revised the language, formatting, figures, tables, and statements throughout the manuscript.

Below, we provide a point-by-point response to each comment.

Reviewer #1: The abstract has a generally clear structure. However, the descriptions of performance are currently mostly qualitative (e.g., “significantly outperforms the baseline model”). The authors should briefly list one or two key quantitative metrics to strengthen the argument that the proposed model outperforms the baseline model.

A1-1:

Thank you for the helpful suggestion. In the revised manuscript, we have added representative quantitative metrics to the Abstract, specifically, we have changed the original phrasing from “AMP-Hunter significantly outperformed baseline models in both AMP classification and MIC prediction.” to “AMP-Hunter outperformed baseline models in both AMP classification and MIC prediction, achieving 95.82% accuracy and a 95.80% F1 score on the test set for classification, and an R² of 0.9245 with an MAE of 0.2305 for MIC prediction.” to more clearly demonstrate the performance improvement

2. When discussing prior studies related to antimicrobial peptide classification and generation, the description is somewhat vague. The authors should cite several representative models or studies in the text, briefly introduce them, and discuss their limitations, thereby providing a more comprehensive overview of the relevant work in this field.

A1-2:

We sincerely thank you for this valuable suggestion. We agree that a clearer introduction to representative studies on AMP classification and generation would help present a more comprehensive overview of the current research landscape and better motivate the rationale of our work. In the revised manuscript, we have expanded the relevant background discussion by adding brief introductions to several representative AMP-related studies. In particular, we added representative studies related to peptide discovery and safety-oriented peptide screening, including the mutual information-based meta-learning framework for bioactive peptide discovery and HLPpred-Fuse for hemolytic peptide prediction, to provide a broader and more balanced overview of current deep learning strategies relevant to AMP identification and design in line 88-92 and 110-114. Specifically, for AMP identification and prediction, we added discussion of representative models such as AMPlify in the line 76-79, the attentive deep learning classifier, and the recent explainable framework in the line 79-83, which together reflect recent advances in AMP classification, activity prediction, and potency-oriented screening, and TP-LMMSG in the line 85-88, which used the graph attention trasnformer model in the classification task. For AMP generation, we added representative studies including PepVAE in the line 90-96, the deep generative framework based on the VAE and combined with the MIC predictor to generated better AMP sequences, and HydrAMP in the line 96-100, which illustrate recent progress in latent-space peptide design, activity-guided generation, and potency optimization.

3. When introducing the generator method, the description of the “MIC-guided optimization” component is unclear, and its implementation requires a more explicit explanation. The authors should clarify whether the MIC value serves as an optimization objective (e.g., a loss function) to guide the training and generation processes during MIC-guided evolution, or whether it is used as a selection criterion to enhance the transparency of the methodology.

A1-3:

We are grateful for this important comment. In AMP-Forge, the predicted MIC is used as the training objective for the predictor, a guidance signal for latent space optimization, and a screening criterion for generated candidate sequences. First, during model training, the MIC value serves as the training target for the predictor, helping it more accurately predict the corresponding MIC value directly from the latent variables. Specifically, the generative model simultaneously learns sequence reconstruction and MIC value prediction during training, with the MIC value incorporated into the total training loss function via a regression loss term. Second, during the sequence generation phase, the learned MIC predictor provides guidance in the latent space, steering the generation toward sequences with lower MIC values. The generated candidate sequences are further evaluated by predicting their MIC values using the trained AMP-Hunter. Sequences predicted to have lower MIC values are prioritized for retention, as lower MIC values indicate stronger antimicrobial efficacy. To improve transparency and consistency, we have revised the corresponding description in abstract part (line 18-19), introduction part (line 144-149) and methods part (line 349-358)

4. In the Results section, the authors mention that the comparative experiments with CNNs can serve as ablation tests for the proposed method. In addition, the authors should include ablation test results comparing the use of simple embeddings instead of ESM2 embeddings, as well as the use of a random graph instead of the PAM250 evolutionary graph in the GNN. This would further verify the effectiveness of each core component of the proposed method.

A1-4:

Thank you very much for your valuable and practical suggestions. The original manuscript only included ablation studies regarding the CNN component and it lacked experiments to verify the contributions of the two core components ESM2-based node feature extraction and the PAM250-based graph construction strategy to the performance of the model. To address this, we conducted additional ablation studies: we replaced the ESM2 embeddings with manually designed sequence descriptors (comprising amino acid composition, dipeptide composition, and physicochemical properties) and substituted the PAM250 evolutionary graph with a graph construction strategy based on cosine similarity. We reasoned that using a random graph representation which preserves no sequence or feature-related neighborhood structure would make it difficult to determine whether the advantages of PAM250 stem from its biologically meaningful similarity definition or merely from graph connectivity itself. In contrast, while the cosine similarity-based approach discards the evolutionary substitution information inherent in PAM250, it still constructs edges based on the similarity of sequence representations. Consequently, this comparison allows us to specifically evaluate whether PAM250 provides added value beyond a generic similarity-based graph.

The new results indicate that both components contribute a lot to the final performance. In the classification task, replacing ESM2-derived node representations with manually designed physicochemical features caused the test set accuracy to drop from 95.82% to 90.34%; replacing the PAM250 graph with the cosine similarity-based strategy resulted in an accuracy of 91.16%. Similarly, in the regression task, these ablation experiments led to performance declines, with the test set Mean Squared Error rising from 0.1523 to 0.7866 and 0.6963, respectively. These results further validate the effectiveness of using ESM2 for node feature extraction and the PAM250 evolutionary biology-informed graph construction strategy employed in AMP-Hunter.

We have incorporated these ablation study results and the associated discussion into the revised "Results" section to more clearly elucidate the contributions of each core component.

Reviewer #2: This manuscript proposes a comprehensive framework for the discovery of antimicrobial peptides (AMPs). The framework integrates sequence-based prediction of potential AMPs and their minimum inhibitory concentration (MIC) values, utilizes MIC predictions to guide the generation of optimized novel AMPs, and employs molecular dynamics simulations for validation. The proposed approach is methodologically rigorous, and by integrating multiple components into a closed-loop workflow, it not only facilitates a streamlined AMP discovery process but also shows strong potential for accelerating antimicrobial peptide design.

The experimental results and supporting analyses are convincing and demonstrate the effectiveness of the proposed framework. Overall, this work presents a promising contribution to the field and is suitable for publication after the authors address the following remarks and comments to further improve the clarity, comprehensibility, and completeness of the manuscript:

a. Novelty and Framework Integration
The manuscript proposes a framework that integrates multiple components; however, the authors do not sufficiently explain how the integration itself forms an innovative and unified framework that distinguishes it from existing AMP discovery workflows. The authors are encouraged to further elaborate on how their framework differs from previous related studies and clarify whether the observed improvements primarily arise from the integration strategy or from the performance of the individual components.

A2-1:

Thank you for the suggestions that help us describe the structure of the paper more clearly. Our proposed framework differs from existing AMP discovery workflows in that this study does not focus on introducing these components independently, but rather couples them into an activity-driven closed-loop workflow. In particular, the MIC predicted by our discriminator, AMP-Hunter, is used as a unified optimization signal throughout the sequence generation, candidate screening, and iterative optimization processes, thereby linking AMP identification and AMP design under a common activity criterion. Furthermore, guided by multi-sequence alignment constraints and MIC values, AMP-Forge preserves the conserved functional fragments identified by multi-sequence alignment while optimizing non-conserved regions. This approach ensures that sequence improvements are controlled rather than resulting from unconstrained de novo exploration. Therefore, this innovative and unified framework does not use these components sequentially; rather, it passes the output of one module such as the MIC value predicted by AMP-Hunter as a clear decision signal to the next module, such as the training module in AMP-Forge, to train a predictor mapping from latent variables to MIC values. Furthermore, the same prediction and screening criteria are consistently applied throughout the entire process, from the prediction to the generation and on to the downstream screening. Regarding whether the observed improvements primarily arise from the integration strategy or from the performance of individual components, the newly added ablation experiments for AMP-Hunter show that the improvement is not attributable to a single module alone. In the classification task, removing NCGCN reduced the test ACC and F1 from 95.82% and 95.80% to 94.54% and 94.38%, while removing CNN caused a much larger drop to 86.25% and 85.72%. Replacing ESM2-derived node representations with handcrafted sequence descriptors or replacing the PAM250-based graph with a cosine similarity-based graph also led to clear performance degradation, with test ACC values decreasing to 90.34% and 91.16%, respectively. A similar trend was observed in MIC regression, where the full AMP-Hunter achieved the best test performance (MSE = 0.1523, R² = 0.9245), whereas all ablated variants showed substantial deterioration. These results indicate that the final performance of AMP-Hunter arises from the complementary integration of local sequence pattern extraction, graph-based relational learning, biologically informative node representations, and evolution-aware graph construction, rather than from a single dominant component.

b. Relationship Between Challenges and Proposed Solutions
The study outlines several challenges associated with current deep learning methods and proposes corresponding solutions. However, the relationship between each specific challenge and its associated solution is not yet clearly articulated. The authors should consider clarifying how each proposed component directly addresses the corresponding limitation in order to better demonstrate the effectiveness and rationale of the overall framework.

A2-2:

We greatly appreciate this valuable feedback. To improve the clarity and logical coherence of the relationship between existing challenges and corresponding solutions, we have revised the relevant descriptions in the introduction to more clearly articulate the correspondence between each limitation in existing AMP prediction and generation methods and the specific design choices in our framework. Specifically, we have modified the introduction in lines 161 to 188 of the introduction, first describe the problem addressed by the contribution before introducing the contribution itself, in order to strengthen the connection and logical coherence between existing issues in the field and the improvements offered by this study and the corresponding components.

c. Evaluation of Generated Sequences
The generated sequences are presented in Table 9; however, the evidence supporting their claimed superiority remains somewhat limited. Including a more systematic comparison of physicochemical properties would help substantiate the claimed improvements and provide a clearer basis for evaluating the quality and effectiveness of the generated peptides.

A2-3:

We sincerely appreciate your valuable suggestion. The original manuscript did indeed lack predictions and analyses of relevant physicochemical properties, as well as comparative analyses with sequences from the original dataset. In the revised manuscript, we have added predictions for the relevant physicochemical properties of the generated sequences in Table 15(due to revisions to the manuscript, Table 9 has been updated to Table 15) using the built-in prediction tools from the DBAASP database, which are consistent with the prediction tools used in the original manuscript. In addition, we have further included a comparative analysis of the generated peptides with reference sequences from the original dataset across different length ranges to strengthen the argument that the generated sequences possess superior quality and effectiveness compared to the original sequences. Furthermore, to ensure better coherence between the added analysis of the physicochemical properties of the generated sequences and the preceding and following content, we have moved the selection criteria for the relevant generated sequences and the revised Table 15 from the “Molecular Dynamics Simulation Parameter Settings” section in the original manuscript to the “Comparative Analysis of Physicochemical Properties and Predicted MIC Values” section. The corresponding text revisions are located from line 857 to line 873.

d. Biological Interpretability of Generated Peptides
Although the study includes feature-space comparisons between the generated sequences and sequences from the original datasets, the biological interpretability of the generated peptides remains relatively limited. Additional sequence-level analyses, such as motif conservation, residue composition analysis, or functional pattern comparisons, would provide deeper insight into whether the generated peptides retain biologically meaningful characteristics.

A2-4:

Thank you very much for your suggestion. By adding motif analysis of the generated peptides and the original peptides, we can more comprehensively demonstrate that beyond the existing analysis of feature space distributions that the generated peptides are not merely random peptides that happen to resemble the original peptides in the feature space, but rather retain biological plausibility. Following your suggestion, we have added a sequence-level comparison of motifs and amino acid patterns. We compared the generated peptides with length-matched subsets from the original peptide dataset and plotted amino acid distribution maps for both the original and generated peptides, as well as a map of amino acid properties for the 11 generated peptide sequences selected through screening. The results indicate that the generated peptides not only retain the enrichment of hydrophobic and positively charged residues, as well as AMP-like sequence organization patterns, but also exhibit sequence variability compared to the original peptides. Specific modifications are located at line 752-775, and the motif analysis figure is the newly added Fig 4.

e. Discussion of Limitations
While the proposed framework demonstrates promising performance, the manuscript would benefit from a more critical discussion of its limitations. Such discussion would help contextualize the findings, clarify the boundaries of applicability, and better inform future research directions and practical applications.

A2-5:

We appreciate your constructive feedback, which has helped us evaluate our work more comprehensively. To provide a more thorough and objective description and assessment of this study, we have expanded the discussion section in the revised manuscript to offer a more balanced evaluation of the method’s scope and limitations. Specifically, we have addressed the following points: the relatively limited nature of the current dataset, which may restrict the generalizability of this method to broader classes of antimicrobial peptides; the fact that the current workflow is primarily computational, meaning that the inferred advantages in efficacy, selectivity, and safety still require validation through systematic experiments; and the limitations arising from the limited simulation time. We have also revised the concluding remarks to reflect the contributions and limitations of this study and have outlined the focus of future work. The relevant revisions are located from line 1171-1192.

Reviewer #3: This manuscript presents a computational framework for antimicrobial peptide discovery by integrating an AMP discriminator, AMP-Hunter, with an MIC-guided generator, AMP-Forge. The overall goal of combining AMP classification, MIC prediction, sequence generation, physicochemical property screening, and molecular dynamics simulation is interesting and potentially useful. The reported classification and regression performance appears promising, and the idea of using predicted MIC values to guide peptide generation is relevant to potency-oriented AMP design. However, the current manuscript still has several important issues that need to be addressed before the technical validity and reproducibility of the work can be properly assessed.
1. The manuscript requires substantial revision in language, formatting, and presentation. There are many grammatical, typographical, and formatting issues throughout the manuscript. For example, the subsection title “Datasetsx” appears to be a typographical error, “RMSE” is written as “RSME” in the regression tables, and the Data Availability statement contains grammatically incorrect wording such as “The data used by are available”. In addition, the main text contains several non-native expressions, incomplete sentences, and awkward phrases that make the technical description difficult to follow. These issues reduce the professionalism of the manuscript and may obscure the authors’ intended meaning. The authors should thoroughly revise the language and carefully check all section titles, tables, figure captions, equations, algorithms, and statements for consistency and accuracy.

A3-1:

Thank you for your detailed and constructive feedback. The original manuscript contained several issues related to grammar, typography, formatting, and presentation, which detracted from the clarity of the work. In the revised version, we have thoroughly proofread the entire text and made comprehensive revisions to the language and formatting of all sections.

Specifically, we have corrected the typographical error in the subsection title “Datasetsx” that you mentioned; the “RMSE” in the regression table, which was incorrectly written as “RSME”; grammatical errors in the data availability statement, such as “The data used by are available”; as well as typographical and formatting errors in tables, figure captions, algorithms, equations, and the data availability statement. In addition, we have revised numerous sentences that were non-native in expression or awkwardly phrased, as well as incomplete sentences, subject-verb agreement issues, tense inconsistencies, punctuation errors, and inconsistencies in terminology to improve readability and accuracy. Furthermore, we have checked the consistency of figure and table citations, unit names, capitalization conventions, and symbol notation throughout the manuscript. The revised version is now more polished and easier to understand.

2. The MIC-guided generation logic contains a critical inconsistency. The proposed framework is based on the premise that lower MIC values indicate stronger antimicrobial activity. Therefore, the generator should be optimized toward sequences with lower predicted MIC values. However, Algorithm 2 states that candidate sequences are retained when MICr > MIC0, whereas Figure 1 and the main text indicate that sequences with MIC < MIC0 are retained. This is a serious inconsistency because it directly concerns the optimization objective of AMP-Forge. The authors should clarify whether this is a typographical error in the pseudocode or whether the implementation actually used this condition. The direction of MIC optimization must be made consistent across the algorithm, equations, figure, textual description, and implementation.

A3-2:

Thank you very much for your careful review of the sections regarding MIC value optimization in the algorithm and main text, and for raising this important point. After carefully reviewing the manuscript and the actual implementation code, we have confirmed that the condition ‘MICr> MIC_0_’ in Algorithm 2 was a typographical error in the pseudocode. The actual implementation in AMP-Forge is consistently optimized to yield lower predicted MIC values, which aligns with the biological premise that the lower the MIC value, the stronger the antimicrobial activity.

In the revised manuscript, we have corrected Algorithm 2 to ensure that candidate sequences are retained only when their predicted MIC values are lower than the initial value (MICr < MIC_0_). Furthermore, we have carefully revised the relevant statements in the figure caption of Figure 1 and the corresponding text, including the Methods and Results sections, to ensure that the pseudocode, figure captions, methodological descriptions, and narrative explanations are fully consistent regarding the optimization direction, thereby making the relevant statements clearer and less prone to ambiguity and confusion. Furthermore, we have clarified the role of MIC in AMP-Forge. During the generative model training phase, the difference between the predicted MIC and the actual value serves as a component of the loss function, training the MIC predictor to directly predict MIC values from latent variables with greater accuracy; during the generation phase, it functions both as the optimization objective for latent space search and as the evaluation criterion for candidate sequence ranking and resampling.

These corrections do not affect previously reported results or conclusions, as the inconsistencies were limited to the pseudocode descriptions and did not affect the actual implementation. The relevant revisions are located from line 430-434, 463-465, 486-488, 566-568 and in Algorithm 2.

3. Several key implementation details of graph construction and NC grouping are unclear. The manuscript states that PAM250 is used to compute pairwise similarities between peptide sequences and construct the graph, but it does not explain how PAM250-based similarity is calculated for sequences of different lengths. It is also unclear what threshold τ was used for graph construction, how this threshold was selected, and how sensitive the model performance is to this choice. In addition, the NC-based grouping strategy needs clearer explanation. The authors should specify whether NC grouping uses ground-truth labels, predicted labels, or MIC values, and how NC is computed for validation and test samples. The manuscript should also clarify whether the graph is constructed using the full dataset or only the training set. If the graph is built before data splitting or includes validation/test nodes during message passing, there may be a risk of information leakage. These details are essential for evaluating the reliability and reproducibility of the reported results.

A3-3:

Thank you very much for this important comment. The additions and clarifications regarding the implementation details of graph construction and NC grouping in the manuscript, along with the analysis of threshold sensitivity, will help readers better understand this work. In the revised manuscript, we have clarified that pairwise sequence similarity is calculated using the Smith-Waterman local alignment with the PAM250 substitution matrix and normalized using the geometric mean of the self-alignment scores to account for differences in sequence length. Additionally, we have explicitly stated that the graph construction threshold is set to 0.2 and clarified the graph-construction procedure and added a sensitivity analysis of 𝜏 under the same training-validation-test split for both the classification and regression tasks. Specifically, we evaluated 𝜏 values from 0.1 to 0.9. For the classification task, 𝜏 = 0.2 gave the best overall performance, with validation accuracy of 96.65% and test accuracy of 95.88%, outperforming all other tested thresholds. In contrast, thresholds farther from 0.2 led to clear performance degradation, especially on the test set. These results support 𝜏= 0.2 as the most appropriate choice for the classification graph. For the regression task, performance was comparatively stable across a moderate range of thresholds, but 𝜏= 0.2 achieved the best validation performance overall, while also maintaining strong test performance. Therefore, we selected 𝜏= 0.2 based on validation performance and overall consistency across tasks. At the same time, the results also show that the performance of the model is not very sensitive to the choice of 𝜏. We have now added the exact threshold value, the rationale for selecting it in the revised manuscript , and the corresponding sensitivity results in the Appendix (S1-S4 Table.pdf). Furthermore, we have provided additional details regarding the NC grouping strategy that training nodes retain their true labels, while non-training nodes use model-predicted labels when updating the neuron group mask. The graph was constructed using only sequence-derived features and PAM250-based similarity scores, without using any class labels or MIC values. During optimization, supervised loss was computed exclusively on training nodes. The NC mask was updated using forced labels for training nodes and predicted labels for non-training nodes. Specifically, after each update step, ground-truth labels were retained for training samples, whereas validation and test samples were assigned their current predicted labels before recomputing the NC-based partition. Therefore, validation and test labels were never used in graph construction or parameter optimization. The related revisions are located from line 214-218, 256-263.

4. The description of AMP-Hunter as a multi-task model is not sufficiently rigorous. The manuscript repeatedly refers to AMP-Hunter as a multi-task discriminator for AMP classification and MIC prediction. However, the current description does not make clear whether classification and regression are trained jointly in a single model or separately using the same feature extraction architecture. If this is a true multi-task learning model, the authors should provide the joint loss function, explain how the classification and regression losses are weighted, and describe whether the two task heads are optimized simultaneously. If the two tasks are trained independently, then the term “multi-task learning” should be used more cautiously or revised. At present, the training strategy is ambiguous and may mislead readers about the actual model design.

A3-4:

Thank you very much for raising this important point. After carefully reviewing the workflow of the AMP-Hunter classifier, we have made corresponding adjustments to the description in the revised manuscript. The two related prediction tasks included in AMP-Hunter (namely, AMP classification and MIC regression) utilize a shared CNN-NCGCN feature extraction backbone, but these two tasks are trained independently rather than jointly.

Specifically, the classification and regression tasks share the same feature extraction framework, but their output layers and loss functions differ. The classification task employs a task-specific output layer optimized using cross-entropy loss, while the regression task uses a task-specific output layer optimized using mean squared error loss. The current implementation does not utilize a joint loss function or loss weighting strategy, nor does it optimize these two tasks simultaneously. To avoid misleading readers, we have revised the relevant descriptions throughout the manuscript and replaced ambiguous terms such as “multi-task discriminator” and “multi-task model” with the more accurate term “shared-architecture discriminator,” as well as using equivalent task-specific descriptions where appropriate. Specifically, we have modified all instances of “multi-task” terminology in the manuscript, including the abstract, the relevant section of the caption for Figure 1, the framework description of AMP-Hunter in Section 2.3 “Feature Fusion of CNN and NCGCN for Antimicrobial Peptides Prediction,” as well as the results and discussion sections pertaining to multi-task aspects, to ensure consistency and transparency in the description of the model design and training strategy.

5. The experimental validation is not sufficient to support the contribution of each proposed module. Although the classification and regression results are encouraging, the manuscript lacks systematic ablation studies. The proposed framework includes multiple components, such as ESM2 embeddings, PAM250-based graph construction, NCGCN, CNN feature extraction, MIC guidance, MSA-based masking, and Monte Carlo resampling. However, the current experiments do not clearly show how much each component contributes to the final performance. Treating the CNN baseline as an indirect ablation is not sufficient. The authors should conduct explicit ablation experiments by removing or replacing key modules, such as ESM2, the PAM250 graph, NCGCN, CNN, MIC guidance, MSA masking, and Monte Carlo resampling. Without these experiments, it is difficult to determine whether the improvement comes from the proposed framework as a whole or mainly from one dominant component.

A3-5:

Thank you very much for your valuable and practical suggestions. To explicitly evaluate the contribution of each core component in AMP-Hunter, we conducted ablation experiments by removing or replacing key modules in the discriminator. Specifically, four ablated variants were constructed: without NCGCN, in which the graph-based NCGCN branch was removed; without CNN, in which the CNN branch was removed; Handcrafted features instead of ESM2, in which ESM2-derived node representations were replaced with handcrafted sequence descriptors composed of amino acid composition, dipeptide composition, and physicochemical properties; and Cosine graph instead of PAM250 graph, in which the PAM250-based graph construction strategy was replaced with cosine similarity-based graph construction. The performance of these variants on the AMP classification and MIC regression tasks is summarized in Tables 7–10.

The ablation results show that all components contribute to the final performance. For AMP classification, the full AMP-Hunter model consistently achieved the best performance on both the validation and test sets. Removing the NCGCN module caused a moderate but consistent decrease in performance, with the test ACC and F1 declining from 95.82% and 95.80% to 94.54% and 94.38%, respectively. Removing the CNN branch caused the most substantial deterioration, reducing the test ACC and F1 to 86.25% and 85.72%, respectively. Replacing ESM2-derived node features with handcrafted sequence descriptors or replacing the PAM250-based graph with a cosine similarity-based graph also led to marked performance degradation, with test ACC values decreasing to 90.34% and 91.16%, respectively. A similar trend was observed in the MIC regression task. The full AMP-Hunter model achieved the best overall performance. Removing the NCGCN resulted in a performance drop; removing the CNN branch caused the most severe performance degradation. Similarly, replacing the ESM2-derived representations with manually designed sequence descriptors, or replacing the PAM250-based graph construction with a cosine similarity-based graph construction, both led to significant performance declines. Overall, these ablation experiment results indicate that the performance of AMP-Hunter stems from the complementary integration of CNN-based local feature extraction, NCGCN-based graph representation learning, ESM2-derived node features, and PAM250-guided graph construction, rather than relying solely on any single component. We have added the corresponding ablation tables and discussion in the revised Results section.

To evaluate the effect of these components on sequence generation, we compared the full AMP-Forge model with three ablated variants, each model was used to generate 5,000 peptide sequences. The generated sequences were assessed using sequence-level and activity-oriented metrics, including uniqueness, diversity, novelty, similarity to known AMPs, and predicted MIC.

The results show that the full AMP-Forge achieved the best overall balance, with the highest uniqueness, high diversity, moderate novelty, retained similarity to known AMPs, and the lowest predicted MIC. Removing MIC guidance increased the predicted MIC and reduced both uniqueness and novelty, indicating that explicit MIC-oriented optimization is important for generating less redundant and more active candidates. Removing Monte Carlo resampling caused the most severe reduction in uniqueness and also produced the worst predicted MIC, suggesting that this strategy is important for controlling redundancy and stabilizing candidate selection during optimization. Removing MSA masking increased novelty but reduced similarity to known AMPs and markedly worsened predicted MIC, indicating that unconstrained sequence exploration may move further away from known AMP patterns at the cost of predicted potency and biological plausibility.

Taken together, these results show that the improvement of AMP-Forge does not arise from a single dominant component alone. Instead, MIC guidance, Monte Carlo resampling, and MSA-based masking play complementary roles in potency-oriented optimization, candidate selection, and preservation of biologically meaningful sequence constraints, respectively. We have added the corresponding ablation table and discussion in the revised Results section and also the description of the mentioned sequence-level metrics to evaluate the performance of the proposed generator in the revised “3.2. Performance Evaluation Metrics”. Therefore, the revised manuscript now provides explicit ablation evidence for both AMP-Hunter and AMP-Forge, which more clearly supports the contribution of the major components in the proposed framework.

6. The generation task needs stronger benchmarking and more appropriate evaluation. The manuscript does not provide direct comparisons with representative peptide or AMP generation methods, such as HydrAMP, PepVAE, or other relevant generative models. As a result, it is difficult to judge whether AMP-Forge offers a meaningful advantage over existing methods. In addition, the manuscript argues that novelty and diversity metrics are not suitable because the generation process preserves conserved fragments. This explanation is not fully convincing. Even for motif-preserving or template-guided generation, novelty, diversity, and sequence similarity to known AMPs remain important for evaluating whether the model generates genuinely useful candidates rather than near-duplicates of the training sequences. The authors should either include appropriate novelty and diversity analyses or provide a much stronger justification for excluding these metrics.

A3-6:

We are grateful for your important comment. In the revised manuscript, we therefore added direct comparisons between AMP-Forge and several representative peptide generation models, including Diffusion, GAN, LSTM, VAE, HydrAMP, ProGen, and AMPGEN. For fair comparison, each model was used to generate 5,000 peptide sequences, and all generated sets were evaluated using uniqueness, diversity, novelty, similarity to known AMPs, and predicted MIC.

The results show that AMP-Forge achieved the best predicted MIC among all compared models, while also maintaining high uniqueness, high diversity, and a moderate level of novelty and similarity to known AMPs. These results indicate that AMP-Forge does not simply generate near-duplicates of known AMPs, nor does it drift excessively far from known AMP space. Instead, it achieves a favorable balance between potency-oriented optimization and sequence-level quality.

We also revised the manuscript to clarify that rather than excluding novelty and diversity metrics, we now explicitly state that, for a constrained and MIC-oriented generator AMP-Forge which preserves conserved fragments through multiple sequence alignment and then performs local optimization in the unmatched regions, novelty and diversity are important supplementary evaluation metrics but should not be treated as the sole optimization objectives. This is because maximizing novelty alone may produce sequences that deviate excessively from biologically meaningful AMP patterns, whereas preserving excessive similarity to known AMPs may reduce the value of generation. We have added the benchmarking results and the corresponding discussion in the revised Results section.

7. Some claims based on molecular dynamics simulations should be stated more cautiously. The MD simulations provide useful in silico evidence that selected peptides may interact with bacterial membrane models and induce local membrane perturbation. However, MD simulations alone cannot confirm actual antimicrobial activity, safety, stability, or therapeutic potential. Claims such as “confirmed antimicrobial potential” or “safer and more stable” should therefore be softened unless supported by experimental MIC assays, hemolysis assays, cytotoxicity tests, serum stability tests, or other wet-lab validation. The authors should clearly distinguish computational prediction from experimental validation.

A3-7:

Thank you for raising this important point. We agree that molecular dynamics simulations should be regarded as computational evidence rather than experimental validation. Therefore, in the revised manuscript, we have amended all statements that might imply definitive validation of antimicrobial activity, safety, stability, or therapeutic potential. Specifically, we have replaced terms such as “confirm,” “validate,” and “safer and more stable” with more cautious phrasing, such as “suggest,” “support,” and “provide computational evidence.” We have also clarified that molecular dynamics simulations are intended to complement computational prediction and screening phases, rather than replace experimental minimum inhibitory concentration (MIC), hemolysis, cytotoxicity, or serum stability assays. These revisions have been incorporated into the “Abstract,” “Author’s Summary,” “Results,” and “Discussion” sections, respectively.

Reviewer #4: This paper addresses the critical issue of efficient antimicrobial peptide discovery by proposing a closed-loop computational framework that integrates generation, screening, and validation, thereby offering a solution to the growing public health threat posed by rising antibiotic resistance. The authors systematically integrate sequence features derived from protein large language models with features from graph neural networks and convolutional neural networks. They organically combine these with a MIC-guided sequence generation strategy and perform multi-level validation of candidate sequences through screening based on physicochemical properties and molecular dynamics simulations. The generated peptides selected in this manner demonstrate antibacterial potential. Overall, this work demonstrates good systematicity in method integration and task design, capable of simultaneously addressing antimicrobial prediction, activity prediction, and sequence optimization within a unified framework, exhibiting a certain degree of innovation and application potential. Furthermore, the authors conducted a relatively comprehensive analysis of the physicochemical properties and membrane interaction behavior of the generated sequences, thereby enhancing the interpretability of the results at the biological level.

The manuscript is well written and the work is well connected to previous research in the field， but there is room for further improvement in the explanation of the framework and the biological discussion. The following specific comments are offered to further enhance the clarity of the paper.

Firstly, the “generation-screening-validation” closed-loop system proposed in this paper is highly systematic and represents a key highlight of the article. In particular, the use of MIC as a unified objective spanning both generation and screening provides the overall workflow with a clear direction for optimization. However, the information dependencies and functional boundaries between the various modules remain somewhat unclear at present. Readers may still be confused about how AMP-Hunter and multiple sequence alignment collaborate during the generation process.

A4-1:

Thank you for this insightful comment. To address the issue that the interaction between AMP-Hunter and MSA in the generation process were not sufficiently clear, we revised both the Introduction and Method sections to more explicitly clarify the roles and information flow between modules in the proposed “generation–screening–validation” framework. Specifically, we now explain that MSA is used to preserve evolutionarily conserved antimicrobial functional fragments and constrain the sequence search space, while AMP-Hunter serves as an MIC-guided discriminator that evaluates generated candidates and provides optimization feedback during latent-space refinement. We further clarified how these two components collaborate to balance biological consistency and antimicrobial optimization during sequence generation. The relevant revisions are located from line 140-152 and line 439-443.

Secondly, this paper employs a generation strategy based on multi-sequence alignment to preserve conserved regions combined with local optimization, which effectively avoids the instability associated with fully random generation, making it highly valuable for practical applications. At the same time, this strategy implies that the generation process relies to some extent on the existing sequence space; therefore, its strengths lie more in “optimizing existing patterns” rather than fully exploring new spaces. Could the authors provide a thorough explanation of the applicability of the method and offer a more comprehensive evaluation of this strategy?

A4-2:

Thank you very much for your feedback, which helps us clarify the applicability of our proposed method. The generation strategy we propose is an optimization framework that preserves conservation, rather than an unrestricted de novo sequence generator. In the revised manuscript, we have clarified the scope and limitations of AMP-Forge in the subsection first introducing the generator and in the discussion section. Specifically, we further elaborate in the manuscript that the proposed generation method aims to optimize antimicrobial peptides by preserving conserved regions identified through multiple sequence alignment and introducing MIC-guided local evolution in variable regions. We also provide a comprehensive and objective analysis showing that while this generation strategy enhances stability and feasibility for practical screening, it also implies more limited exploration of potential novel patterns. Furthermore, by combining sequence-level motif analysis, feature space overlap, and physicochemical property comparisons, we evaluated whether this generation method captures the biological rationality of the original antimicrobial peptides. We verified that AMP-Forge primarily optimizes biologically meaningful AMP motifs while simultaneously improving activity-related properties. The relevant revisions are located from line 337-342 and line 1150-1161.

Thirdly, the paper systematically demonstrates the advantages of the generated peptides in terms of charge, hydrophobicity, amphiphilicity, and membrane interaction behavior in the analysis of physicochemical property and MD simulations, which is very well-founded. However, the current biological interpretation focuses primarily on explaining the classical AMP mechanism, with limited discussion on whether new design principles or potential mechanisms emerge. To more fully demonstrate the characteristics of generated peptides across different length ranges, the authors can analyze and present whether different optimization strategies emerge for different length ranges.

A4-3:

Thank you very much for this detailed suggestion, which has allowed us to conduct a more in-depth analysis of the physicochemical characteristics and potential mechanisms of action of the generated peptides in different length groups, building upon our comprehensive evaluation of the physicochemical properties and membrane-interaction mechanisms of the generated peptides. In the revised manuscript, we have expanded Section 3.9, “Comparative Analysis of Physicochemical Properties and Predicted MIC Values,” to include an analysis of the differences in physicochemical properties among the different length groups and to provide a length-based biological interpretation of the generated peptides, laying the groundwork for subsequent discussions on the distinct mechanisms of action among the different length groups. In the results section 3.11. Membrane Interaction Analysis of Selected Peptides, we analyzed the differences in membrane interaction mechanisms among peptides of different length groups, summarized the mechanisms of action for the generated peptides, and concluded that the optimization patterns for different length groups are not consistent. Shorter peptides tend to exhibit a stronger enrichment in charge-related features and compact amphiphilic characteristics, whereas medium- and longer-length peptides more frequently display optimization patterns associated with increased structural stability, expanded peptide-membrane contact surfaces, and deeper membrane disruption. These observations indicate that this study provides practical, length-dependent design principles for the activity-oriented optimization of antimicrobial peptides. The relevant revisions are located from line 843-856 and line 940-949.

Lastly, this paper integrates existing AMP database resources and combines them with MIC information for classification and regression tasks, while also applying filtering criteria based on sequence length and amino acid composition. This provides a well-structured data foundation for model training. However, from the perspectives of method reproducibility and result interpretability, the current description of the data source integration and filtering process remains somewhat brief.

A4-4:

We appreciate your constructive feedback. The original manuscript provided only a brief description of the integration and filtering of data sources. In the revised version, we have provided additional details regarding the organization and processing of the datasets in the “Datasets” subsection of the Results section. Specifically, we have expanded the description of the datasets to clarify the sources of the original studies, their application in specific tasks such as AMP classification, MIC regression, and sequence generation, as well as the filtering criteria based on peptide length and sequence composition. The relevant revisions are located from line 502-524.

Overall, the closed-loop AMP design framework proposed in this paper demonstrates a high level of performance in terms of method integration, performance metrics, and multi-level verification, and holds significant potential for application. By addressing the suggestions and comments mentioned above, the paper will be more comprehensive and possess greater academic rigor.

We again sincerely thank the editors and reviewers for your careful assessment and valuable suggestions. We believe that the revised manuscript has been substantially improved in response to these comments, and we hope that it is now suitable for publication in PLOS Computational Biology.

Sincerely,

Wenyu Zhang, Yizheng Wang, Yixiao Zhai, Pinglu Zhang, Yijie Ding*, Quan Zou*
