# Supplementary material for: A unified framework for potency-oriented AMP discovery via multi-modal learning and guided sequence synthesis
Source: PLoS Comput Biol. 2026 Sep 3;22(9):e1014771. doi: 10.1371/journal.pcbi.1014771 (PMC13568523; doi:10.1371/journal.pcbi.1014771)
Supplement: Peer review history

# Peer review: Decision Letter 0

**DOI:** 10.1371/journal.pcbi.1014771.r001

**Authors:** Pedro Mendes, Ju Xiang

13 May 2026

A Unified Framework for Potency-Oriented AMP Discovery via Multi-Modal Learning and Guided Sequence Synthesis

PLOS Computational Biology

Dear Dr. Zhang,

Thank you for submitting your manuscript to PLOS Computational Biology. After careful consideration, we feel that it has merit but does not fully meet PLOS Computational Biology's publication criteria as it currently stands. Therefore, we invite you to submit a revised version of the manuscript that addresses the points raised during the review process.

Please submit your revised manuscript by Jul 13 2026 11:59PM. If you will need more time than this to complete your revisions, please reply to this message or contact the journal office at ploscompbiol@plos.org. When you're ready to submit your revision, log on to https://www.editorialmanager.com/pcompbiol/ and select the 'Submissions Needing Revision' folder to locate your manuscript file.

Please include the following items when submitting your revised manuscript:

* A letter that responds to each point raised by the editor and reviewer(s). You should upload this letter as a separate file labeled 'Response to Reviewers'. This file does not need to include responses to formatting updates and technical items listed in the 'Journal Requirements' section below.

* A marked-up copy of your manuscript that highlights changes made to the original version. You should upload this as a separate file labeled 'Revised Manuscript with Track Changes'.

* An unmarked version of your revised paper without tracked changes. You should upload this as a separate file labeled 'Manuscript'.

We look forward to receiving your revised manuscript.

Kind regards,

Ju Xiang, Ph.D.

Academic Editor

PLOS Computational Biology

Pedro Mendes

Section Editor

PLOS Computational Biology

**Additional Editor Comments:**

The reviewers have raised several constructive comments and suggestions regarding the clarity, methodology, data analysis, and presentation of your manuscript, which we believe will help improve the quality and rigor of your work. We invite you to carefully address each comment raised by the reviewers and revise your manuscript accordingly.

**Journal Requirements:**

1) Please ensure that the CRediT author contributions listed for every co-author are completed accurately and in full.

At this stage, the following Authors/Authors require contributions: Wenyu Zhang, Yizheng Wang, Yixiao Zhai, Pinglu Zhang, Yijie Ding, and Quan Zou. Please ensure that the full contributions of each author are acknowledged in the "Add/Edit/Remove Authors" section of our submission form.

The list of CRediT author contributions may be found here: https://journals.plos.org/ploscompbiol/s/authorship#loc-author-contributions

2) Please upload all main figures as separate Figure files in .tif or .eps format. For more information about how to convert and format your figure files please see our guidelines:

https://journals.plos.org/ploscompbiol/s/figures

3) Please amend your detailed Financial Disclosure statement. This is published with the article. It must therefore be completed in full sentences and contain the exact wording you wish to be published.

- State the initials, alongside each funding source, of each author to receive each grant. For example: "This work was supported by the National Institutes of Health (####### to AM; ###### to CJ) and the National Science Foundation (###### to AM)."

- State what role the funders took in the study. If the funders had no role in your study, please state: "The funders had no role in study design, data collection and analysis, decision to publish, or preparation of the manuscript.".

If you did not receive any funding for this study, please simply state: u201cThe authors received no specific funding for this work.u201d

**Reviewers' comments:**

Reviewer's Responses to Questions

**Comments to the Authors:**

**Please note here if the review is uploaded as an attachment.**

Reviewer #1: he abstract has a generally clear structure. However, the descriptions of performance are currently mostly qualitative (e.g., “significantly outperforms the baseline model”). The authors should briefly list one or two key quantitative metrics to strengthen the argument that the proposed model outperforms the baseline model.

2. When discussing prior studies related to antimicrobial peptide classification and generation, the description is somewhat vague. The authors should cite several representative models or studies in the text, briefly introduce them, and discuss their limitations, thereby providing a more comprehensive overview of the relevant work in this field.

3. When introducing the generator method, the description of the “MIC-guided optimization” component is unclear, and its implementation requires a more explicit explanation. The authors should clarify whether the MIC value serves as an optimization objective (e.g., a loss function) to guide the training and generation processes during MIC-guided evolution, or whether it is used as a selection criterion to enhance the transparency of the methodology.

4. In the Results section, the authors mention that the comparative experiments with CNNs can serve as ablation tests for the proposed method. In addition, the authors should include ablation test results comparing the use of simple embeddings instead of ESM2 embeddings, as well as the use of a random graph instead of the PAM250 evolutionary graph in the GNN. This would further verify the effectiveness of each core component of the proposed method.

Reviewer #2: This manuscript proposes a comprehensive framework for the discovery of antimicrobial peptides (AMPs). The framework integrates sequence-based prediction of potential AMPs and their minimum inhibitory concentration (MIC) values, utilizes MIC predictions to guide the generation of optimized novel AMPs, and employs molecular dynamics simulations for validation. The proposed approach is methodologically rigorous, and by integrating multiple components into a closed-loop workflow, it not only facilitates a streamlined AMP discovery process but also shows strong potential for accelerating antimicrobial peptide design.

The experimental results and supporting analyses are convincing and demonstrate the effectiveness of the proposed framework. Overall, this work presents a promising contribution to the field and is suitable for publication after the authors address the following remarks and comments to further improve the clarity, comprehensibility, and completeness of the manuscript:

a. Novelty and Framework Integration

The manuscript proposes a framework that integrates multiple components; however, the authors do not sufficiently explain how the integration itself forms an innovative and unified framework that distinguishes it from existing AMP discovery workflows. The authors are encouraged to further elaborate on how their framework differs from previous related studies and clarify whether the observed improvements primarily arise from the integration strategy or from the performance of the individual components.

b. Relationship Between Challenges and Proposed Solutions

The study outlines several challenges associated with current deep learning methods and proposes corresponding solutions. However, the relationship between each specific challenge and its associated solution is not yet clearly articulated. The authors should consider clarifying how each proposed component directly addresses the corresponding limitation in order to better demonstrate the effectiveness and rationale of the overall framework.

c. Evaluation of Generated Sequences

The generated sequences are presented in Table 9; however, the evidence supporting their claimed superiority remains somewhat limited. Including a more systematic comparison of physicochemical properties would help substantiate the claimed improvements and provide a clearer basis for evaluating the quality and effectiveness of the generated peptides.

d. Biological Interpretability of Generated Peptides

Although the study includes feature-space comparisons between the generated sequences and sequences from the original datasets, the biological interpretability of the generated peptides remains relatively limited. Additional sequence-level analyses, such as motif conservation, residue composition analysis, or functional pattern comparisons, would provide deeper insight into whether the generated peptides retain biologically meaningful characteristics.

e. Discussion of Limitations

While the proposed framework demonstrates promising performance, the manuscript would benefit from a more critical discussion of its limitations. Such discussion would help contextualize the findings, clarify the boundaries of applicability, and better inform future research directions and practical applications.

Reviewer #3: This manuscript presents a computational framework for antimicrobial peptide discovery by integrating an AMP discriminator, AMP-Hunter, with an MIC-guided generator, AMP-Forge. The overall goal of combining AMP classification, MIC prediction, sequence generation, physicochemical property screening, and molecular dynamics simulation is interesting and potentially useful. The reported classification and regression performance appears promising, and the idea of using predicted MIC values to guide peptide generation is relevant to potency-oriented AMP design. However, the current manuscript still has several important issues that need to be addressed before the technical validity and reproducibility of the work can be properly assessed.

1. The manuscript requires substantial revision in language, formatting, and presentation. There are many grammatical, typographical, and formatting issues throughout the manuscript. For example, the subsection title “Datasetsx” appears to be a typographical error, “RMSE” is written as “RSME” in the regression tables, and the Data Availability statement contains grammatically incorrect wording such as “The data used by are available”. In addition, the main text contains several non-native expressions, incomplete sentences, and awkward phrases that make the technical description difficult to follow. These issues reduce the professionalism of the manuscript and may obscure the authors’ intended meaning. The authors should thoroughly revise the language and carefully check all section titles, tables, figure captions, equations, algorithms, and statements for consistency and accuracy.

2. The MIC-guided generation logic contains a critical inconsistency. The proposed framework is based on the premise that lower MIC values indicate stronger antimicrobial activity. Therefore, the generator should be optimized toward sequences with lower predicted MIC values. However, Algorithm 2 states that candidate sequences are retained when MICr > MIC0, whereas Figure 1 and the main text indicate that sequences with MIC < MIC0 are retained. This is a serious inconsistency because it directly concerns the optimization objective of AMP-Forge. The authors should clarify whether this is a typographical error in the pseudocode or whether the implementation actually used this condition. The direction of MIC optimization must be made consistent across the algorithm, equations, figure, textual description, and implementation.

3. Several key implementation details of graph construction and NC grouping are unclear. The manuscript states that PAM250 is used to compute pairwise similarities between peptide sequences and construct the graph, but it does not explain how PAM250-based similarity is calculated for sequences of different lengths. It is also unclear what threshold τ was used for graph construction, how this threshold was selected, and how sensitive the model performance is to this choice. In addition, the NC-based grouping strategy needs clearer explanation. The authors should specify whether NC grouping uses ground-truth labels, predicted labels, or MIC values, and how NC is computed for validation and test samples. The manuscript should also clarify whether the graph is constructed using the full dataset or only the training set. If the graph is built before data splitting or includes validation/test nodes during message passing, there may be a risk of information leakage. These details are essential for evaluating the reliability and reproducibility of the reported results.

4. The description of AMP-Hunter as a multi-task model is not sufficiently rigorous. The manuscript repeatedly refers to AMP-Hunter as a multi-task discriminator for AMP classification and MIC prediction. However, the current description does not make clear whether classification and regression are trained jointly in a single model or separately using the same feature extraction architecture. If this is a true multi-task learning model, the authors should provide the joint loss function, explain how the classification and regression losses are weighted, and describe whether the two task heads are optimized simultaneously. If the two tasks are trained independently, then the term “multi-task learning” should be used more cautiously or revised. At present, the training strategy is ambiguous and may mislead readers about the actual model design.

5. The experimental validation is not sufficient to support the contribution of each proposed module. Although the classification and regression results are encouraging, the manuscript lacks systematic ablation studies. The proposed framework includes multiple components, such as ESM2 embeddings, PAM250-based graph construction, NCGCN, CNN feature extraction, MIC guidance, MSA-based masking, and Monte Carlo resampling. However, the current experiments do not clearly show how much each component contributes to the final performance. Treating the CNN baseline as an indirect ablation is not sufficient. The authors should conduct explicit ablation experiments by removing or replacing key modules, such as ESM2, the PAM250 graph, NCGCN, CNN, MIC guidance, MSA masking, and Monte Carlo resampling. Without these experiments, it is difficult to determine whether the improvement comes from the proposed framework as a whole or mainly from one dominant component.

6. The generation task needs stronger benchmarking and more appropriate evaluation. The manuscript does not provide direct comparisons with representative peptide or AMP generation methods, such as HydrAMP, PepVAE, or other relevant generative models. As a result, it is difficult to judge whether AMP-Forge offers a meaningful advantage over existing methods. In addition, the manuscript argues that novelty and diversity metrics are not suitable because the generation process preserves conserved fragments. This explanation is not fully convincing. Even for motif-preserving or template-guided generation, novelty, diversity, and sequence similarity to known AMPs remain important for evaluating whether the model generates genuinely useful candidates rather than near-duplicates of the training sequences. The authors should either include appropriate novelty and diversity analyses or provide a much stronger justification for excluding these metrics.

7. Some claims based on molecular dynamics simulations should be stated more cautiously. The MD simulations provide useful in silico evidence that selected peptides may interact with bacterial membrane models and induce local membrane perturbation. However, MD simulations alone cannot confirm actual antimicrobial activity, safety, stability, or therapeutic potential. Claims such as “confirmed antimicrobial potential” or “safer and more stable” should therefore be softened unless supported by experimental MIC assays, hemolysis assays, cytotoxicity tests, serum stability tests, or other wet-lab validation. The authors should clearly distinguish computational prediction from experimental validation.

Reviewer #4: This paper addresses the critical issue of efficient antimicrobial peptide discovery by proposing a closed-loop computational framework that integrates generation, screening, and validation, thereby offering a solution to the growing public health threat posed by rising antibiotic resistance. The authors systematically integrate sequence features derived from protein large language models with features from graph neural networks and convolutional neural networks. They organically combine these with a MIC-guided sequence generation strategy and perform multi-level validation of candidate sequences through screening based on physicochemical properties and molecular dynamics simulations. The generated peptides selected in this manner demonstrate antibacterial potential. Overall, this work demonstrates good systematicity in method integration and task design, capable of simultaneously addressing antimicrobial prediction, activity prediction, and sequence optimization within a unified framework, exhibiting a certain degree of innovation and application potential. Furthermore, the authors conducted a relatively comprehensive analysis of the physicochemical properties and membrane interaction behavior of the generated sequences, thereby enhancing the interpretability of the results at the biological level.

The manuscript is well written and the work is well connected to previous research in the field， but there is room for further improvement in the explanation of the framework and the biological discussion. The following specific comments are offered to further enhance the clarity of the paper.

Firstly, the “generation-screening-validation” closed-loop system proposed in this paper is highly systematic and represents a key highlight of the article. In particular, the use of MIC as a unified objective spanning both generation and screening provides the overall workflow with a clear direction for optimization. However, the information dependencies and functional boundaries between the various modules remain somewhat unclear at present. Readers may still be confused about how AMP-Hunter and multiple sequence alignment collaborate during the generation process.

Secondly, this paper employs a generation strategy based on multi-sequence alignment to preserve conserved regions combined with local optimization, which effectively avoids the instability associated with fully random generation, making it highly valuable for practical applications. At the same time, this strategy implies that the generation process relies to some extent on the existing sequence space; therefore, its strengths lie more in “optimizing existing patterns” rather than fully exploring new spaces. Could the authors provide a thorough explanation of the applicability of the method and offer a more comprehensive evaluation of this strategy?

Thirdly, the paper systematically demonstrates the advantages of the generated peptides in terms of charge, hydrophobicity, amphiphilicity, and membrane interaction behavior in the analysis of physicochemical property and MD simulations, which is very well-founded. However, the current biological interpretation focuses primarily on explaining the classical AMP mechanism, with limited discussion on whether new design principles or potential mechanisms emerge. To more fully demonstrate the characteristics of generated peptides across different length ranges, the authors can analyze and present whether different optimization strategies emerge for different length ranges.

Lastly, this paper integrates existing AMP database resources and combines them with MIC information for classification and regression tasks, while also applying filtering criteria based on sequence length and amino acid composition. This provides a well-structured data foundation for model training. However, from the perspectives of method reproducibility and result interpretability, the current description of the data source integration and filtering process remains somewhat brief.

Overall, the closed-loop AMP design framework proposed in this paper demonstrates a high level of performance in terms of method integration, performance metrics, and multi-level verification, and holds significant potential for application. By addressing the suggestions and comments mentioned above, the paper will be more comprehensive and possess greater academic rigor.

**********

**Have the authors made all data and (if applicable) computational code underlying the findings in their manuscript fully available?**

The PLOS Data policy requires authors to make all data and code underlying the findings described in their manuscript fully available without restriction, with rare exception (please refer to the Data Availability Statement in the manuscript PDF file). The data and code should be provided as part of the manuscript or its supporting information, or deposited to a public repository. For example, in addition to summary statistics, the data points behind means, medians and variance measures should be available. If there are restrictions on publicly sharing data or code —e.g. participant privacy or use of data from a third party—those must be specified.

Reviewer #1: None

Reviewer #2: Yes

Reviewer #3: Yes

Reviewer #4: None

**********

PLOS authors have the option to publish the peer review history of their article (what does this mean?). If published, this will include your full peer review and any attached files.

If you choose “no”, your identity will remain anonymous but your review may still be made public.

**Do you want your identity to be public for this peer review?**  For information about this choice, including consent withdrawal, please see our Privacy Policy.

Reviewer #1: No

Reviewer #2: **Yes:** RENZHI CAO

Reviewer #3: No

Reviewer #4: No

[NOTE: If reviewer comments were submitted as an attachment file, they will be attached to this email and accessible via the submission site. Please log into your account, locate the manuscript record, and check for the action link "View Attachments". If this link does not appear, there are no attachment files.]

**Figure resubmission:**

After uploading your figures to PLOS’s NAAS tool - https://ngplosjournals.pagemajik.ai/artanalysis, NAAS will process the files provided and display the results in the "Uploaded Files" section of the page as the processing is complete. If the uploaded figures meet our requirements (or NAAS is able to fix the files to meet our requirements), the figure will be marked as "fixed" above. If NAAS is unable to fix the files, a red "failed" label will appear above. When NAAS has confirmed that the figure files meet our requirements, please download the file via the download option, and include these NAAS processed figure files when submitting your revised manuscript.

**Reproducibility:**

?>

# Author comment: Author response to Decision Letter 1

**DOI:** 10.1371/journal.pcbi.1014771.r002

1 Jul 2026

Attachment Submitted filename: Response to Reviewers.docx

# Peer review: Decision Letter 1

**DOI:** 10.1371/journal.pcbi.1014771.r003

**Authors:** Pedro Mendes, Ju Xiang

28 Aug 2026

Dear Wenyu Zhang Zhang,

We are pleased to inform you that your manuscript 'A Unified Framework for Potency-Oriented AMP Discovery via Multi-Modal Learning and Guided Sequence Synthesis' has been provisionally accepted for publication in PLOS Computational Biology.

Before your manuscript can be formally accepted you will need to complete some formatting changes, which you will receive in a follow up email. A member of our team will be in touch with a set of requests.

Please note that your manuscript will not be scheduled for publication until you have made the required changes, so a swift response is appreciated.

IMPORTANT: The editorial review process is now complete. PLOS will only permit corrections to spelling, formatting or significant scientific errors from this point onwards. Requests for major changes, or any which affect the scientific understanding of your work, will cause delays to the publication date of your manuscript.

Should you, your institution's press office or the journal office choose to press release your paper, you will automatically be opted out of early publication. We ask that you notify us now if you or your institution is planning to press release the article. All press must be co-ordinated with PLOS.

Thank you again for supporting Open Access publishing; we are looking forward to publishing your work in PLOS Computational Biology.

Best regards,

Ju Xiang, Ph.D.

Academic Editor

PLOS Computational Biology

Pedro Mendes

Section Editor

PLOS Computational Biology

***********************************************************

We have reviewed your revised manuscript and responses. All reviewers have consented to the acceptance of this manuscript. The manuscript is hereby recommended to be accepted for publication.

Reviewer's Responses to Questions

**Comments to the Authors:**

**Please note here if the review is uploaded as an attachment.**

Reviewer #1: The author has answered all the questions; I have no further queries.

Reviewer #2: Authors answer all the concerns and the quality of the paper is highly improved, so I would recommend to accept it

Reviewer #3: no

Reviewer #4: The authors have addressed all my concerns. Therefore, I recommend this paper for publication.

**********

**Have the authors made all data and (if applicable) computational code underlying the findings in their manuscript fully available?**

The PLOS Data policy requires authors to make all data and code underlying the findings described in their manuscript fully available without restriction, with rare exception (please refer to the Data Availability Statement in the manuscript PDF file). The data and code should be provided as part of the manuscript or its supporting information, or deposited to a public repository. For example, in addition to summary statistics, the data points behind means, medians and variance measures should be available. If there are restrictions on publicly sharing data or code —e.g. participant privacy or use of data from a third party—those must be specified.

Reviewer #1: None

Reviewer #2: Yes

Reviewer #3: Yes

Reviewer #4: None

**********

PLOS authors have the option to publish the peer review history of their article (what does this mean?). If published, this will include your full peer review and any attached files.

If you choose “no”, your identity will remain anonymous but your review may still be made public.

**Do you want your identity to be public for this peer review?**  For information about this choice, including consent withdrawal, please see our Privacy Policy.

Reviewer #1: No

Reviewer #2: No

Reviewer #3: No

Reviewer #4: No

# Editor report: Acceptance letter

**DOI:** 10.1371/journal.pcbi.1014771.r004

**Authors:** Pedro Mendes, Ju Xiang

PCOMPBIOL-D-26-00846R1

A Unified Framework for Potency-Oriented AMP Discovery via Multi-Modal Learning and Guided Sequence Synthesis

Dear Dr Zhang,

I am pleased to inform you that your manuscript has been formally accepted for publication in PLOS Computational Biology. Your manuscript is now with our production department and you will be notified of the publication date in due course.

The corresponding author will soon be receiving a typeset proof for review, to ensure errors have not been introduced during production. Please review the PDF proof of your manuscript carefully, as this is the last chance to correct any errors. Please note that major changes, or those which affect the scientific understanding of the work, will likely cause delays to the publication date of your manuscript.

Soon after your final files are uploaded, unless you have opted out, the early version of your manuscript will be published online. The date of the early version will be your article's publication date. The final article will be published to the same URL, and all versions of the paper will be accessible to readers.

For Research, Software, and Methods articles, you will receive an invoice from PLOS for your publication fee after your manuscript has reached the completed accept phase. If you receive an email requesting payment before acceptance or for any other service, this may be a phishing scheme. Learn how to identify phishing emails and protect your accounts at https://explore.plos.org/phishing.

Thank you again for supporting PLOS Computational Biology and open-access publishing. We are looking forward to publishing your work!

With kind regards,

Sharmila Kamatchi

PLOS Computational Biology | Carlyle House, Carlyle Road, Cambridge CB4 3DN | United Kingdom ploscompbiol@plos.org | Phone +44 (0) 1223-442824 | ploscompbiol.org | @PLOSCompBiol
